# Elevated Kir2.1/nuclear N2ICD defines a highly malignant subtype of non-WNT/SHH medulloblastomas

**DOI:** 10.1038/s41392-022-00890-7

**Published:** 2022-03-11

**Authors:** Yan-Xia Wang, Haibo Wu, Yong Ren, Shengqing Lv, Chengdong Ji, Dongfang Xiang, Mengsi Zhang, Huimin Lu, Wenjuan Fu, Qing Liu, Zexuan Yan, Qinghua Ma, Jingya Miao, Ruili Cai, Xi Lan, Bin Wu, Wenying Wang, Yinhua Liu, Dai-Zhong Wang, Mianfu Cao, Zhicheng He, Yu Shi, Yifang Ping, Xiaohong Yao, Xia Zhang, Peng Zhang, Ji Ming Wang, Yan Wang, Youhong Cui, Xiu-Wu Bian

**Affiliations:** 1 Institute of Pathology and Southwest Cancer Center, Southwest Hospital, Army Medical University (former Third Military Medical University), 400038 Chongqing, China; 2 Department of Pathology, The First Affiliated Hospital of University of Science and Technology of China, 230036 Hefei, Anhui China; 3 Intelligent Pathology Institute, Division of Life Sciences and Medicine, University of Science and Technology of China, 230036 Hefei, Anhui China; 4 Department of Pathology, General Hospital of Central Theater Command of PLA, 627 Wuluo Road, Hongshan District, 430070 Wuhan, Hubei China; 5 Xinqiao Hospital, Army Medical University, 400038 Chongqing, China; 6 Department of Pathology, The First Affiliated Hospital of Wannan Medical College, 241001 Wuhu, Anhui China; 7 Department of Pathology, Taihe Hospital, Hubei University of Medicine, 442000 Shiyan, Hubei China; 8 Laboratory of Cancer and Immunometabolism, Center for Cancer Research, National Cancer Institute at Frederick, Frederick, MD 21703 US

**Keywords:** CNS cancer, Drug development

## Abstract

Medulloblastoma (MB) is one of the most common childhood malignant brain tumors (WHO grade IV), traditionally divided into WNT, SHH, Group 3, and Group 4 subgroups based on the transcription profiles, somatic DNA alterations, and clinical outcomes. Unlike WNT and SHH subgroup MBs, Group 3 and Group 4 MBs have similar transcriptomes and lack clearly specific drivers and targeted therapeutic options. The recently revised WHO Classification of CNS Tumors has assigned Group 3 and 4 to a provisional non-WNT/SHH entity. In the present study, we demonstrate that Kir2.1, an inwardly-rectifying potassium channel, is highly expressed in non-WNT/SHH MBs, which promotes tumor cell invasion and metastasis by recruiting Adam10 to enhance S2 cleavage of Notch2 thereby activating the Notch2 signaling pathway. Disruption of the Notch2 pathway markedly inhibited the growth and metastasis of Kir2.1-overexpressing MB cell-derived xenograft tumors in mice. Moreover, Kir2.1^high^/nuclear N2ICD^high^ MBs are associated with the significantly shorter lifespan of the patients. Thus, Kir2.1^high^/nuclear N2ICD^high^ can be used as a biomarker to define a novel subtype of non-WNT/SHH MBs. Our findings are important for the modification of treatment regimens and the development of novel-targeted therapies for non-WNT/SHH MBs.

## Introduction

Medulloblastoma (MB) is one of the most common malignant brain tumors of childhood arising from either the cerebellum or brainstem^1–3^ with an incidence ranging from 0.20 to 0.58 cases per 100,000 persons.^4^ Although current therapeutic regimens enable patients to achieve a survival rate of ~70%, the survivors often have severe lifelong motor and cognitive defects^5–7^ and up to one-third of them may develop recurrence.^8^ Each treatment, such as maximal surgical resection, craniospinal irradiation, and chemotherapy, is accompanied by complications.^9^ Therefore, a better understanding of MB pathogenesis and refining the risk-based stratification may avoid overtreatment or undertreatment of the disease.

MB has been the subject of intense investigations for several decades and substantial progresses have been made,^10–12^ in particular, comprehensive genomics and transcriptomics have greatly advanced the understanding of the molecular mechanisms underlying tumorigenesis and progression of MB, thereby facilitating molecular classifications. Based on the genetic and transcriptomic profiles, MB is divided into four subgroups: WNT, SHH, Group 3, and Group 4.^2,13^ The WNT and SHH subgroups are characterized with hyperactivation of WNT and SHH pathways, respectively, and both may benefit from targeted therapies, while Group 3 and Group 4 MBs are defined via clustering algorithms without a clear understanding of key signaling pathways and therapeutic targets.^14^ In view of the similar transcriptomes in Group 3 and Group 4 MBs and the lack of known drivers and key signaling pathways, recent revision of WHO Classification of CNS Tumors has assigned Group 3 and Group 4 MBs to a provisional non-WNT/SHH entity.^2,15^ The consensus subgrouping has become the core of the current subclassification of MBs. However, substantial heterogeneity of intra-subgroup of MBs is emerging and each subgroup is further divided into variant subtypes. Using unsupervised class discovery based on 428 MBs profiled by DNA-methylation array, Schwalbe et al. divided MBs into seven molecular subtypes, in which WNT remained unchanged, SHH was split into infant and childhood subtypes, and Group 3 and Group 4 each was split into high- and low-risk subtypes.^16^ By using similarity network fusion (SNF), an integrative analysis of 763 MBs profiled by both gene expression and DNA-methylation array identified 12 different MB subtypes, including two WNT (α and β), four SHH (α, β, γ, and δ), three Group 3 (α, β, and γ), and three Group 4 (α, β, and γ).^17^ Since Group 3 and Group 4 account for over two-thirds of all MB cases and contain more heterogeneous clinical characteristics with worse patient survival than WNT and SHH groups,^18,19^ Northcott et al*.* analyzed these subgroups as an entity with t-Distributed Stochastic Neighbor Embedding (t-SNE) method and identified eight subtypes (I–VIII),^20^ with results further supported by analysis with multiple complementary bioinformatics.^21^ Thus, current molecular classifications of MBs have potent clinical relevance as each subgroup or subtype manifests different clinical features and outcomes.^22,23^ However, much is desired for a better understanding of the molecular heterogeneity of MBs for refined molecular subclassification.

Metastasis is a major cause of poor outcome and a treatment challenge for MB patients.^24,25^ About 45% of Group 3 and 40% of Group 4 MB patients have metastasis at the time of diagnosis, higher than the rate in patients with WNT or SHH subgroup tumors.^19,22,25^ Hence, identification of markers/drivers/pathways involved in the metastasis of Group 3 and Group 4 (non-WNT/SHH) MBs is important for refining their subtypes and developing targeted therapy. In recent years, the role of ion channels in the progress of MB has received attention. For example, Huang et al*.* found that potassium channel EAG2 promotes growth and metastasis of MB; Francisco et al*.* reported that Chloride intracellular channel 1 (CLIC1) cooperates with EAG2 to promote MB growth.^26–28^ Moreover, Valdora et al*.*, through mRNA expression profiling of 64 primary tumor samples, identified inwardly-rectifying potassium channel J2 (KCNJ2/Kir2.1) as one of the most upregulated genes on chromosome 17q in tumors with 17q gain, and suggested that Kir2.1 could be used as a marker of poor prognosis and a therapeutic target in non-WNT/SHH MBs.^29^ In our previous study of gastric cancer, Kir2.1 was found to significantly enhance the invasion and metastasis of gastric cancer cells by interacting with STK38. These lead to our hypothesis that Kir2.1 may also be involved in the metastasis of non-WNT/SHH MBs.

In this study, we report that Kir2.1 is preferentially expressed by non-WNT/SHH MBs and contributes to tumor progression by recruiting disintegrin and metalloproteinase domain-containing protein 10 (Adam10) to enhance S2 cleavage of Notch2, thereby activating the Notch2 pathway. Adam10 inhibitor markedly diminished the growth of orthotopic xenograft tumors derived from non-WNT/SHH MB cells and prolonged the lifespan of tumor-bearing mice. Patients with Kir2.1^high^/high nuclear Notch2 intracellular domain (Kir2.1^high^/nN2ICD^high^) MBs had the worst outcomes compared to patients with tumors containing other expression patterns of Kir2.1 and nN2ICD. Our findings, therefore, define Kir2.1^high^/nN2ICD^high^ as a biomarker for a highly malignant subtype of non-WNT/SHH MBs and suggest the Kir2.1-Notch2 pathway as a novel therapeutic target.

## Results

### Kir2.1 is preferentially expressed in human non-WNT/SHH MBs

**Fig. 1:**
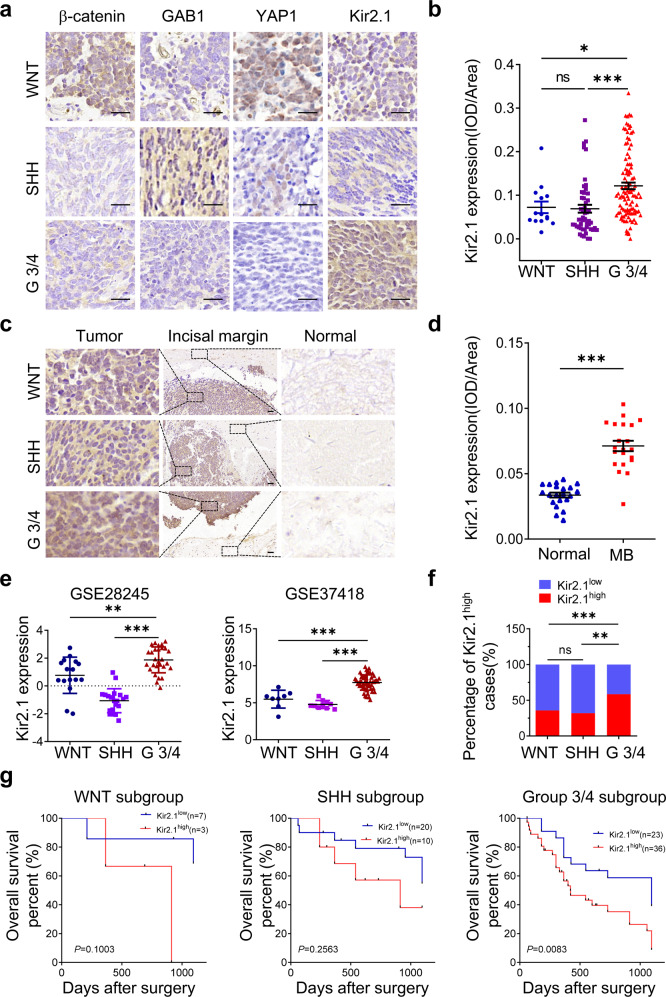
High expression of Kir2.1 in human Group 3/4 MBs. **a** Representative IHC images of Kir2.1 expression in different subgroup MB specimens, where WNT subgroup MBs showing nuclear β-catenin and YAP1 positive, but GAB1 negative; SHH subgroup MBs showing GAB1 and nuclear YAP1 positive, but nuclear β-catenin negative; non-WNT/SHH subgroup MBs showing nuclear β-catenin, nuclear YAP1and GAB1 negative. Scale bar = 25 μm. **b** IHC scores showing significantly higher expression of Kir2.1 in Group 3/4 than in WNT and SHH MBs. Data are shown as mean ± S.D., *n* = 170, ns, not significant, *P < 0.05, ***P < 0.0001, ANOVA test. **c** IHC images showing stronger Kir2.1 staining in tumors than in corresponding normal tissues from the incisal margin. Scale bar = 50 μm. **d** Scatter diagram showing higher IHC scores of Kir2.1 in tumors than in normal tissues. Data are shown as mean ± S.D., *n* = 21, ***P < 0.0001, Student’s *t*-test. **e** Analyses of GEO GES28245 and GES37418 data sets showing significantly higher expression of Kir2.1 in Group 3/4 than in WNT and SHH MBs. Data are shown as the mean ± S.D.; *n* = 64 and 76 for GSE28245 and GSE37418, respectively; **P < 0.001; ***P < 0.0001, ANOVA test. **f** Higher proportion of Kir2.1^high^ cases (Kir2.1 expression levels over median) in Group 3/4 than in WNT and SHH groups. *n* = 170, ns, not significant, **P < 0.001, ***P < 0.0001, Chi-square test. **g** Kaplan–Meier curves based on subgroups showing a negative correlation between the levels of Kir2.1 and the overall survival in Group 3/4 (*P* = 0.0083), but not in WNT (*P* = 0.1003) and SHH (*P* = 0.2563) subgroup MBs, Log-rank test

The levels of Kir2.1 expression in surgical specimens of 170 MB cases were examined by using immunohistochemistry (IHC). Kir2.1 was stained in the membrane and cytoplasm of MB cells with significantly increased detection in non-WNT/SHH than in WNT and SHH subgroup tumors (Fig. 1a, b). In 21 specimens with paired adjacent non-tumor tissues, the expression of Kir2.1 in tumor tissues was significantly higher than in non-tumor tissues (Fig. 1c, d). Analysis of both GSE28245 and GSE37418 from the Gene Expression Omnibus (GEO) database (Supplementary Table 1) also showed that the levels of Kir2.1 expression were markedly higher in non-WNT/SHH MBs than in WNT and SHH subgroup tumors (Fig. 1e). Using the median to define Kir2.1 expression level, a significantly high proportion of Kir2.1^high^ cases was observed in Group 3/4 compared with WNT and SHH groups (Fig. 1f). The expression level of Kir2.1 was positively correlated with age and molecular subgroups, but not with gender and histological subtype in 170 MB cases (Table 1). Subgroup-based Kaplan–Meier analysis showed that high expression of Kir2.1 was associated with poorer overall survival (OS) in non-WNT/SHH MBs, but not in WNT and SHH subgroup MBs (Fig. 1g). These results suggest that Kir2.1 is preferentially expressed in non-WNT/SHH MBs with a poorer prognosis.

**Table 1:** The relationship between Kir2.1 expression and clinicopathological features in MB patients

Characteristics	Kir2.1 expression			
	Total (*n* = 170)	Low or none (*n* = 87)	High (*n* = 83)	*P* value
Gender				0.925
Male	110	56 (50.9%)	54 (49.1%)	
Female	60	31 (51.7%)	29 (48.3%)	
Age (years)				0.111
<3	8	1 (12.5%)	7 (87.5%)	
3–6	36	19 (52.8%)	17 (47.2%)	
7–16	70	40 (57.1%)	30 (42.9%)	
≥17	56	27 (48.2%)	29 (51.8%)	
Histologic subtypes^a^				0.385
Classic	117	65 (55.6%)	52 (44.4%)	
Desmoplastic/extensive nodularity	21	8 (38.1%)	13 (61.9%)	
Anaplastic	9	4 (44.4%)	5 (55.6%)	
Large cell	23	10 (43.5%)	13 (56.5%)	
Molecular subgroups				**0.005**
WNT	14	9 (64.3%)	5 (35.7%)	
SHH	50	34 (68%)	16 (32%)	
Non-WNT/SHH	106	44 (41.5%)	62 (58.5%)	

^a^The four main histologic types of MB recognized by the WHO are classic, desmoplastic/nodular, and MB with extensive nodularity, anaplastic and large cells^2^

*p* values that are statistically significant are shown in bold

### Kir2.1 promotes the invasion, metastasis, and epithelial–mesenchymal transition of non-WNT/SHH MB cells

**Fig. 2:**
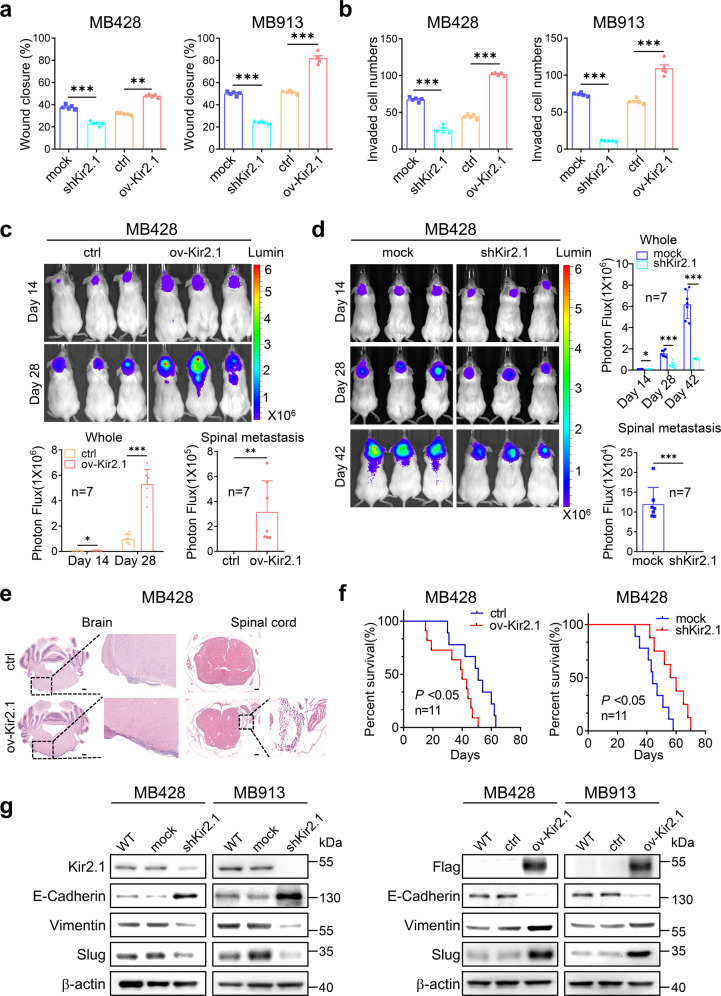
Kir2.1 enhances invasive and metastatic capacities of non-WNT/SHH MB cells. **a** Wound-healing assays showing that silencing Kir2.1 significantly decreased, while overexpressing Kir2.1 significantly increased the migratory ability of MB428 and MB913 non-WNT/SHH MB cells. Data are shown as mean ± S.D., *n* = 5, **P < 0.001, ***P < 0.0001, Student’s *t*-test. **b** Matrigel-transwell invasion assays showing that silencing Kir2.1 significantly decreased, while overexpressing Kir2.1 significantly increased the invasive ability of MB428 and MB913 non-WNT/SHH MB cells. Data are shown as mean ± S.D., *n* = 5, ***P < 0.0001, Student’s *t*-test. **c** Representative bioluminescent images (upper panel) showing higher tumorigenicity and stronger metastatic abilities of Kir2.1-overexpressing MB428 cells than control cells. The lower left panel showing the quantification of photon flux of the whole brain and the lower right panel showing the quantification of photon flux of the spinal cord (metastasis). Data are shown as mean ± S.D., *n* = 7, *P < 0.05, **P < 0.001, ***P < 0.0001, Student’s *t*-test. **d** Representative bioluminescent images (left panel) showing lower tumorigenicity and weaker metastatic abilities of Kir2.1-knockdown MB428 cells than mock cells. The right upper panel showing the quantification of photon flux of the whole brain and the right lower panel showing the quantification of photon flux of the spinal cord (metastasis). Data are shown as mean ± S.D., *n* = 7, ns, not significant, *P < 0.05, ***P < 0.0001, Student’s *t*-test. **e** Representative HE staining of the brain and spinal sections showing more metastatic foci of Kir2.1-overexpressing MB428 cells than control cells. Scale bar = 100 μm. **f** Kaplan–Meier survival curves showing that mice bearing xenograft tumors formed by Kir2.1-overexpressing cells had shorter lifespan than control cells, while mice bearing Kir2.1-knockdown xenograft tumors had a longer lifespan than the mock. *n* = 11, *P* < 0.05, Log-rank test. **g** Western blotting showing that silencing Kir2.1 resulted in upregulation of E-cadherin and downregulation of Vimentin and Slug in MB428 and MB913 cells, while overexpressing Kir2.1 led to downregulation of E-cadherin and upregulation of Vimentin and Slug in MB428 and MB913 cells. Here, anti-Flag antibody was used to bind Flag-tagged Kir2.1 in Kir2.1-overexpressing cells

In 7 MB cell lines or primary MB cells (3 SHH and 4 non-WNT/SHH MB cells), non-WNT/SHH MB cells expressed a higher level of Kir2.1 than SHH subgroup cells (Supplementary Fig. 1). After the establishment of Kir2.1-knockdown and -overexpression cell models, we examined the affection of Kir2.1 on the migration and invasion capabilities of non-WNT/SHH MB cells in vitro. MB428 and MB913 with Kir2.1 knockdown (Supplementary Fig. 2a, b) migrated more slowly into the monolayer scratching wound area compared to cells containing scramble short hairpin RNA (shRNA) mock cells, while MB cells with Kir2.1 overexpression (Supplementary Fig. 2c, d) migrated into the scratching area more rapidly than the control cells (Fig. 2a and Supplementary Fig. 3a). Those MB cells with Kir2.1-knockdown also exhibited decreased invasive capability, while opposite results were obtained from Kir2.1-overexpressing MB cells (Fig. 2b and Supplementary Fig. 3b). Then the tumorigenic, invasive, and metastatic capacities of Kir2.1-overexpressing MB cells and paired control cells were examined by injecting the cells into the cerebellum of NOD/SCID mice. Bioluminescent imaging on days 14 and 28 showed that Kir2.1-overexpressing MB428 and MB913 cells formed larger tumors and stronger spinal cord metastasis than control cells (Fig. 2c and Supplementary Fig. 4a), while Kir2.1-knockdown MB428 cells showed lower tumorigenicity and weaker metastatic abilities of Kir2.1-knockdown MB428 cells than mock cells on day 14, 28, and 42 (Fig. 2d). Moreover, orthotopic tumors derived from Kir2.1-overexpressing MB cells showed more metastatic foci in the brain and spinal area as compared to tumors derived from control cells (Fig. 2e and Supplementary Fig. 4b). IHC staining confirmed that the overexpression and knockdown of Kir2.1 in MB cells were stable in vivo (Supplementary Fig. 4c). The lifespan of mice bearing Kir2.1-overexpressing tumors was also markedly shorter than the control, whereas mice bearing Kir2.1-knockdown tumors had a longer lifespan than the mock (Fig. 2f and Supplementary Fig. 4d). These data indicate that Kir2.1 enhances the metastatic capability of non-WNT/SHH MB cells. In addition, IHC staining showed a high percentage of Ki67 positive cells and a very low percentage of Cleaved Caspase-3 positive cells in xenografts derived from control and mock cells; Overexpression/silencing of Kir2.1 significantly increased/decreased the percentage of Ki67 positive cells, respectively, but had no significant effect on the percentage of Cleaved Caspase-3-positive cells (Supplementary Fig. 4c). These results suggest that Kir2.1 is also involved in the proliferation of non-WNT/SHH MB cells.

EMT is a pivotal event for enhanced invasion and metastasis capabilities of tumor cells.^30,31^ We, therefore, evaluated the involvement of EMT in Kir2.1-promoted invasion and metastasis of non-WNT/SHH MB cells. Knockdown of Kir2.1 resulted in the upregulation of epithelial marker E-cadherin but downregulation of mesenchymal marker Vimentin in MB428 and MB913 cells (Fig. 2g, left panel). In contrast, Kir2.1-overexpression enhanced the EMT phenotype in those cells with decreased E-cadherin but increased Vimentin expression (Fig. 2g, right panel, and Supplementary Fig. 5). Moreover, the level of Slug, a key transcription factor in EMT, was significantly correlated with increased Kir2.1 in both MB428 and MB913 cells (Fig. 2g and Supplementary Fig. 5), implying the involvement of Kir2.1 in EMT of MB cells. These findings further support Kir2.1 as an important contributor to enhance invasion/metastasis and EMT of non-WNT/SHH MB cells.

### Notch2 signaling is associated with the action of Kir2.1 in non-WNT/SHH MB cells

Kir2.1 has been reported to exhibit pro-malignant activity either as a potassium channel^32–34^ or by promoting protein–protein interaction.^35–37^ We, therefore, examined the pro-malignant activity of Kir2.1 first based on its channel function in MB. Treatment of Kir2.1-overexpressing MB428 and MB913 cells with Zacopride, an inward-rectifier potassium current (IK1) stimulator,^38^ potently elevated IK1 in the cells (Supplementary Figs. 6a and 7a), but did not affect their migration and invasion capabilities (Supplementary Figs. 6b, c, 7b, c, and 8). Treatment of these cells with ML133, a specific inhibitor of Kir2.1 channel activity,^39^ completely disrupted IK1 enhanced by Kir2.1 overexpression in those cells (Supplementary Figs. 6d and 7d), but did not alter their migration and invasion capabilities (Supplementary Figs. 6e, f, 7e, f, and 8). Since the extracellular pore-forming region of Kir2.1 serves as the “ion-selectivity filter”, in which mutation of GYG into AAA leads to the loss of potassium channel function,^40^ we introduced Kir2.1 with GYG to AAA (Kir2.1-Mutant, Kir2.1-M) mutation into MB cells. Interestingly, although Kir2.1-M lost its regulatory function on IK1 (Supplementary Figs. 6g and 7g), the mutant reserved comparable activity to promote migration and invasion of MB428 and MB913 cells to the level similar to the cells overexpressing Kir2.1 (Supplementary Figs. 6h, i, 7h, i, and 8). Thus, the pro-malignant activity of Kir2.1 in MB cells was independent of its ion channel function but rather, dependent on a yet unknown protein–protein interaction mechanism.

**Fig. 3:**
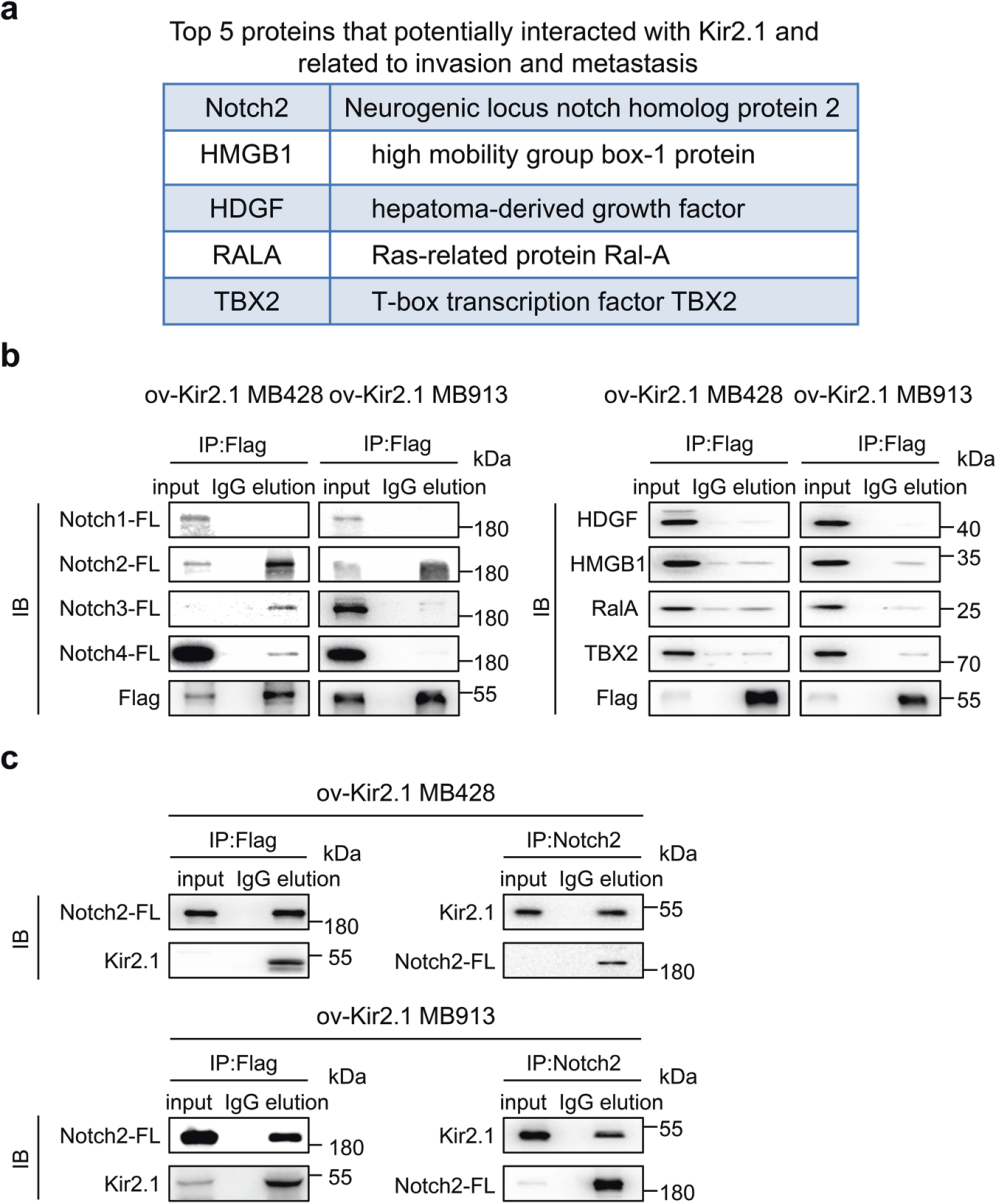
Kir2.1 physically interacts with Notch2 in non-WNT/SHH MB cells. **a** Immunoprecipitation-mass spectrometry-predicted top 5 Kir2.1-interacting proteins related to invasion and metastasis in non-WNT/SHH MB cells. **b** Co-IP showing that Notch2 was physically conjugated with Kir2.1 among the predicated proteins. Since anti-Kir2.1 antibody for IP was unavailable, anti-Flag antibody was used to bind Flag-tagged Kir2.1 in Kir2.1-overexpressing cells. **c** Co-IP validation of the interaction between Notch2-FL and Kir2.1 in MB428 and MB913 cells

Our previous study revealed that in gastric cancer (GC) cells, Kir2.1-promoted cell invasion and metastasis via interacting with serine/threonine-protein kinase 38 (STK38) to enhance MEKK2-MEK1/2 -ERK1/2 -Snail signaling.^41^ This prompted us to examine the effect of kir2.1-overexpression on the MEKK2-MEK1/2-ERK1/2-Snail pathway in MB cells. As shown in Supplementary Fig. 9, Kir2.1 overexpression did not activate the MEK1/2-ERK1/2 pathway, implying the presence of other pathways involved. To address this issue, Kir2.1 immunoprecipitation followed by mass spectrometry identified 77 proteins as potential Kir2.1-binding partners in MB cells. By mining literature, we found that 20 potential interactors among them were related to invasion and metastasis of cancer (Supplementary Table 5). According to the IP-MS score, the top 5 proteins related to the invasion and metastasis were selected as the research objects^42–50^ (Fig. 3a). Subsequent co-immunoprecipitation (Co-IP) confirmed the interaction of Kir2.1 only with Notch2-full length (Notch2-FL) in both ov-Kir2.1 MB428 and MB913 cells which expressed Flag-Kir2.1 (Fig. 3b, c), and this interaction was verified by Co-IP using the anti-Notch2 antibody in wild type MB428 cells which expressed endogenous Kir2.1 (Supplementary Fig. 10). These results suggest the involvement of the Notch2 pathway in the functions of Kir2.1 in non-WNT/SHH MB cells.

### Kir2.1 promotes the activation of Notch2 pathway in non-WNT/SHH MB cells

**Fig. 4:**
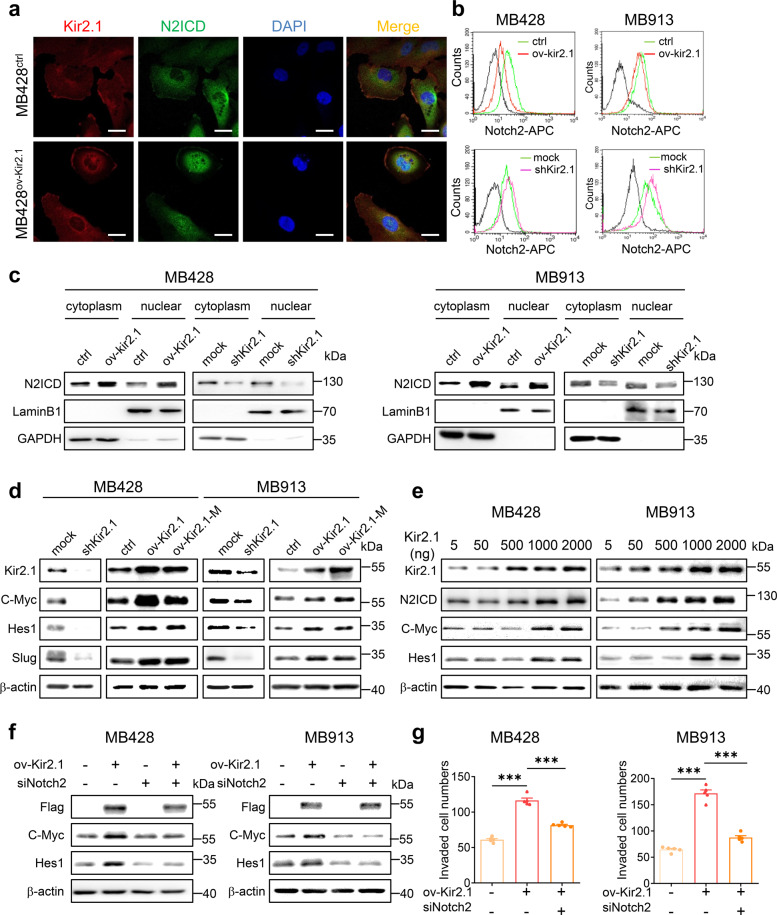
Kir2.1 activates Notch2 signaling in non-WNT/SHH MB cells. **a** Immunofluorescence assays showing that overexpressing Kir2.1enhanced intracellular domain of Notch2 (N2ICD) in the cytoplasm and nuclei in MB428 cells. Scale bar = 20 μm. **b** FACS showing that overexpressing Kir2.1 decreased cell surface Notch2, whereas silencing Kir2.1-enhanced cell surface Notch2 in MB428 and MB913 cells. **c** Western blotting showing that overexpressing Kir2.1-enhanced N2ICD, whereas silencing Kir2.1 reduced N2ICD in both the cytoplasm and nuclei in MB428 and MB913 cells. **d** Western blotting showing that manipulating the expression of Kir2.1 significantly altered the expression of Notch2 pathway-targeted genes *C-Myc*, *Hes1,* and *Slug* in MB428 and MB913 cells. **e** Western blotting showing that transiently transfecting of Kir2.1-overexpressing plasmid altered the expression of Notch2 pathway-targeted genes in dose-dependent manner. **f** Western blotting showing that knockdown of Notch2 attenuated Kir2.1-induced expression of C-Myc and Hes1 in MB428 and MB913 cells. Here, anti-Flag antibody was used to bind Flag-tagged Kir2.1 in Kir2.1-overexpressing cells. **g** Matrigel-transwell invasion assays showing that knockdown of Notch2 attenuated Kir2.1-promoted invasion in MB428 and MB913 cells. Data are shown as the mean ± S.D., *n* = 5, ns, not significant, ***P < 0.0001, ANOVA test

To examine the involvement of the Notch2 pathway in the functions of Kir2.1, we first co-localized Kir2.1 with Notch2 under confocal microscopy in Kir2.1-overexpressing MB428 and control cells, in which Kir2.1 was labeled with red fluorescence while the intracellular domain of Notch2 (N2ICD) was in green. In control MB428 cells, both Kir2.1 and N2ICD were detected mainly in the cell membranes (Fig. 4a, upper panel). However, in Kir2.1-overexpressing MB428 cells, most Notch2 signals were detected in the cytoplasm and nuclei, with the distribution pattern of Kir2.1 unchanged (Fig. 4a, lower panel). It has been known that the maturation and activation of Notch2 include site1 (S1), site2 (S2), and site3 (S3) cleavages.^51^ S2 cleavage releases the extracellular domain of Notch2 (N2ECD) from the membrane, and subsequent S3 cleavage generates functional Notch2 intracellular domain (N2ICD), which translocates to the nucleus as a transcription factor. By using an antibody against the extracellular domain of Notch2 (N2ECD), we detected decreased N2ECD in the membrane of Kir2.1-overexpressing MB cells, but an increase in the cell membrane of Kir2.1-knockdown MB cells as compared with paired control cells (Fig. 4b). We then isolated the cytoplasm and nuclei from Kir2.1-overexpression, -knockdown, and control cells to detect N2ICD by Western blotting. Compared to control cells, Kir2.1 overexpression in MB428 and MB913 cells markedly enhanced N2ICD in both cytoplasm and nuclei, while Kir2.1 knockdown significantly reduced N2ICD in both compartments (Fig. 4c).

To further verify the role of Kir2.1 in Notch2 signaling, we examined the expression of N2ICD target genes, including *C-Myc*, *Hes1*, and *Slug*^52^ in non-WNT/SHH MB cells. As expected, knockdown of Kir2.1 significantly reduced the expression of the three genes, while overexpression of either Kir2.1 or Kir2.1-M potently increased the expression of these genes in non-WNT/SHH MB cells (Fig. 4d). As confirmation, increasing doses of Kir2.1 plasmid transfected into MB428 and MB913 cells progressively enhanced N2ICD production as well as N2ICD target gene expression (Fig. 4e). Furthermore, interfering Notch2 expression in MB428 and MB913 cells with specific siRNA (Supplementary Fig. 11a) reduced the expression of C-Myc and Hes1 originally upregulated by Kir2.1-overexpression (Fig. 4f). Kir2.1-enhanced invasion of MB428 and MB913 cells was also inhibited by Notch2 silencing (Fig. 4g and Supplementary Fig. 11b). Thus, Notch2 participates in the pro-malignant activity of Kir2.1 in non-WNT/SHH MB cells.

### Kir2.1 enhances the S2 cleavage and activation of Notch2 in non-WNT/SHH MB cells

**Fig. 5:**
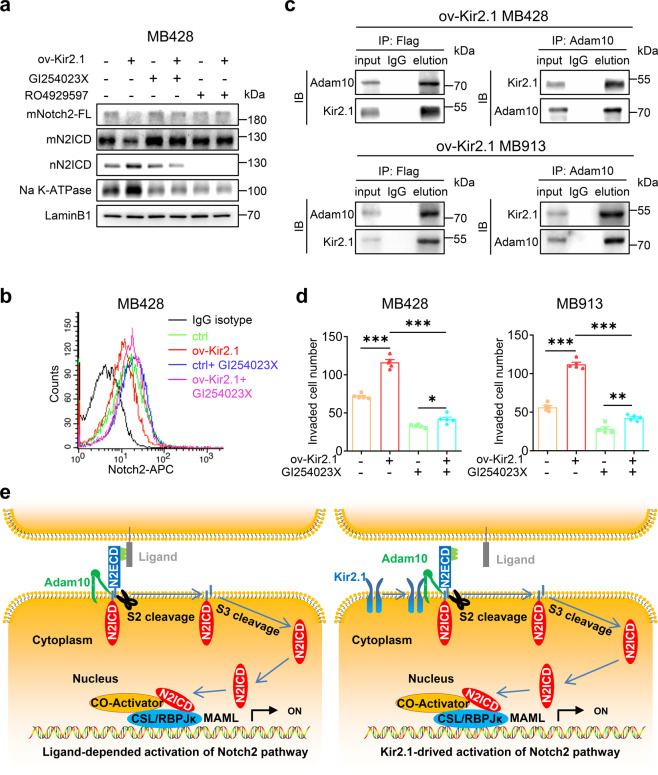
Kir2.1 enhances S2 cleavage to activate Notch2 signaling. **a** Western blotting showing that overexpressing Kir2.1 markedly reduced the membrane full length Notch2 (mNotch2-FL) and the membrane intracellular domain of Notch (mN2ICD), whereas treatment with GI254023X (S2 inhibitor, 3 μM) increased mNotch2-FL and mN2ICD in control cells and abolished the effect of Kir2.1-overexpressing in Kir2.1-overexpressing MB428 cells; treatment with S3 inhibitor (RO4929597) increased mN2ICD and abolished nN2ICD in both Kir2.1-overexpressing MB428 cells and control cells. **b** FACS showing that S2 cleavage inhibitor attenuated the ability of overexpressing Kir2.1 to reduce the extracellular domain of Notch2 in MB428 cells. **c** Co-IP showing Kir2.1 interaction with Adam10. Here, anti-Flag antibody was used to bind Flag-tagged Kir2.1 in Kir2.1-overexpressing cells. **d** Matrigel-transwell invasion assays showing that S2 cleavage inhibitor inhibited Kir2.1-promoted invasion of MB428 and MB913 cells. Data are shown as the mean ± S.D., *n* = 5, ns, not significant, *P < 0.05, **P < 0.001, ***P < 0.0001, ANOVA test. **e** A model summarizing the novel Notch2 signaling pathway activated by Kir2.1. Left panel, S2 cleavage after ligand stimulation in MB cells; Right panel, Kir2.1 triggering S2 cleavage independent of ligand binding in MB cells. N2ECD: Notch2 extracellular domain

The results presented so far suggest the involvement of Kir2.1 in the S2 and/or S3 cleavage of Notch2 in non-WNT/SHH MB cells. To test this hypothesis, we first examined the effect of Adam10, a critical S2 cleavage enzyme, on the activation of Notch2.^53,54^ As shown in Fig. 5a and Supplementary Fig. 12a, overexpressing Kir2.1 in MB cells markedly reduced the full-length membrane Notch2 (mNotch2-FL) (detected by an antibody against the extracellular domain of Notch2) and mN2ICD (N2ICD with a transmembrane domain), but increased nuclear N2ICD (nN2ICD), while treatment with GI254023X, a specific inhibitor of Adam10, abolished the effect of Kir2.1-overexpression, resulting in increased mNotch2-FL and mN2ICD but decreased nN2ICD. Flow cytometer analysis also showed that overexpressing Kir2.1 in MB cells significantly decreased cell surface Notch2-FL as compared to control cells, while GI254023X treatment slightly increased cell surface Notch2-FL in control cells and blocked the effect of Kir2.1 overexpression on decreasing cell surface Notch2-FL in Kir2.1-overexpressing cells (Fig. 5b and Supplementary Fig. 12b). Co-IP showed that Kir2.1 pulled down Adam10 and vice versa in ov-Kir2.1 MB428 and MB913 cells that expressed Flag-Kir2.1 (Fig. 5c), and this interaction was verified by Co-IP using anti-Adam10 antibody in wild type MB428 cells (Supplementary Fig. 13). In addition, treatment with the S2 inhibitor GI254023X markedly decreased the invasive ability of not only Kir2.1-overexpressing but also control MB cells (Fig. 5d and Supplementary Fig. 14). These results suggested that by physical interaction, Kir2.1 recruits Adam10 to participate in the S2 cleavage process of Notch2. Then, we evaluated whether Kir2.1 is involved in the S3 cleavage of Notch2. Although inhibition of γ-secretase, the enzyme complex for S3 cleavage, with RO4929597, increased mN2ICD and abolished nN2ICD both in Kir2.1-overexpressing and control MB cells (Fig. 5a and Supplementary Fig. 12a), Co-IP showed that Kir2.1 did not interact with any component of γ-secretase complex, including Nicastrin, Presenilin1/2, and PEN-2 (Supplementary Fig. 15), implying that γ-secretase complex was not involved in the function of Kir2.1. Thus, Kir2.1 recruits Adam10 to cellular membrane where Notch2 anchors, to enhance S2 cleavage and activation of Notch2 in non-WNT/SHH MB cells (Fig. 5e).

### Kir2.1^high^/nucleus N2ICD^high^ subtype of non-WNT/SHH MB is associated with the poorest patient survival

**Fig. 6:**
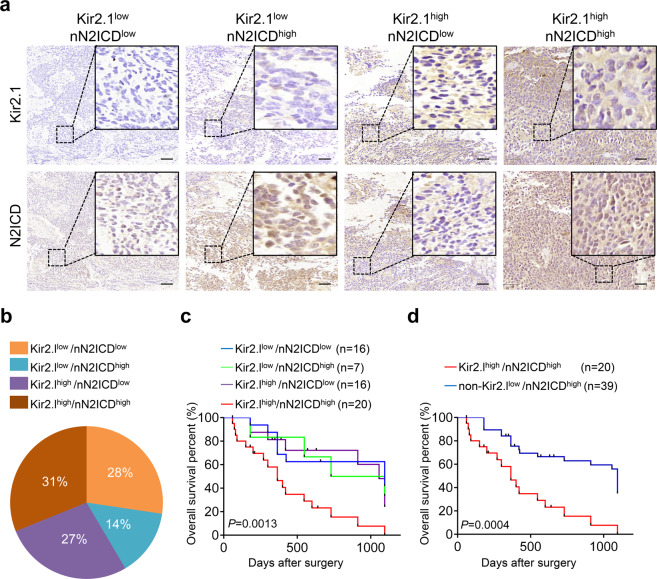
Kir2.1^high^-nN2ICD^high^ defines a novel subtype of non-WNT/SHH MBs with the poorest outcome. **a** Representative IHC images showing the expression status of Kir2.1 and nN2ICD, including Kir2.1^low^/nN2ICD^low^, Kir2.1^low^/nN2ICD^high^, Kir2.1^high^/nN2ICD^low^, and Kir2.1^high^/nN2ICD^high^ in non-WNT/SHH MBs. Scale bar = 100 μm. **b** The percentage of patients with different Kir2.1/nN2ICD expression status in non-WNT/SHH MBs. **c** Kaplan–Meier survival curves showing the poorest outcome of Kir2.1^high^/N2ICD^high^ patients among the patients with different Kir2.1/N2ICD expressing non-WNT/SHH MBs. *P* = 0.0013, Log-rank test. **d** Kaplan–Meier survival curves showing a poorer outcome of the Kir2.1^high^/N2ICD^high^ subtype patients than patients with non-Kir2.1^high^/N2ICD^high^ subtypes in non-WNT/SHH MBs. *P* = 0.0004, Log-rank test

Although the association of Kir2.1^high^ with a poorer OS of patients has suggested that Kir2.1^high^ had a potential as a marker for defining a new subtype of non-WNT/SHH MBs, the elucidation of the mechanism of Kir2.1 action in MB prompted us to consider whether the combination of Kir2.1^high^ with Notch2 activation could more effectively define the subtype of non-WNT/SHH MBs. Therefore, we assessed nN2ICD in 106 non-WNT/SHH MB tumors that underwent Kir2.1 immunostaining (shown in Fig. 1a). We found that high level of nN2ICD was not consistently associated with increased Kir2.1 in the tumors. Based on the expression level of Kir2.1 and nN2ICD, these tumors are divided into 4 subtypes: Kir2.1^low^/nN2ICD^low^ (28%), Kir2.1^low^/nN2ICD^high^ (14%), Kir2.1^high^/nN2ICD^low^ (27%), and Kir2.1^high^/nN2ICD^high^ (31%) (Fig. 6a, b). For 59 patients in the 106-patient cohort with follow-up information, Kaplan–Meier analysis showed that patients with Kir2.1^high^/nN2ICD^high^ had the poorest OS of all subtypes (Fig. 6c). Since the survival curves of the three subtypes excluding Kir2.1^high^/nN2ICD^high^ were similar, we combined these three subtypes into one category and named it non-Kir2.1^high^/nN2ICD^high^ subtypes. Patients with Kir2.1^high^/nN2ICD^high^ subtype showed a significantly shorter lifespan than patients with non-Kir2.1^high^/nN2ICD^high^ MB (Fig. 6d). In addition, the analysis of the relationship between Kir2.1-Notch2 expression in MB tissues and clinicopathological features of the patients showed that Kir2.1^high^/nN2ICD^high^ subtype was positively correlated only with age, an important risk factor in MBs^55,56^ (Table 2). Multivariate analysis also showed that Kir2.1/nN2ICD was an independent factor for OS (Table 3). Therefore, Kir2.1^high^/nN2ICD^high^ act as a biomarker to define a novel malignant subtype of non-WNT/SHH MB in human.

**Table 2:** The relationship between Kir2.1-nN2ICD status and clinicopathological features in non-WNT/SHH MBs

Characteristics	Subtype			
	Overall (*n* = 106)	non-Kir2.1^high^/nN2ICD^high^ (*n* = 73)	Kir2.1^high^/nN2ICD^high^ (*n* = 33)	*P* value
Gender				0.439
Male	76	54 (71.1%)	22 (28.9%)	
Female	30	19 (63.3%)	11 (36.7%)	
Age (years)				**0.038**
<3	6	1 (16.7%)	5 (83.3%)	
3–6	26	19 (73.1%)	7 (26.9%)	
7–16	46	34 (73.9%)	12 (26.1%)	
≥17	28	19 (67.9%)	9 (32.1%)	
Histologic subtypes^a^				0.217
Classic	73	53 (72.6%)	20 (27.4%)	
Non-classic	33	20 (60.6%)	13 (39.4%)	

^a^In our cohort of G 3/4, the number of each histologic subtype MBs were classic *n* = 73, desmoplastic/nodular and MB with extensive nodularity *n* = 18, anaplastic *n* = 0, and large cell *n* = 15. In view of the small number of each subtype excluding classic MB, they were classified into two subtypes, classic subtype (*n* = 73) and non-Classic subtype (*n* = 33), which included the three subtypes excluding classic subtype

*p* values that are statistically significant are shown in bold

**Table 3:** Multivariate analysis of OS in non-WNT/SHH MB patients

Variable	HR	95% CI	*P* value
Gender	1.291	0.646–2.578	0.47
Age (years)	0.804	0.574–1.126	0.204
Histologic subtypes	1.799	0.973–3.324	0.061
Kir2.1/nN2ICD	3.083	1.544–6.154	0.001

### Inactivating Notch2 pathway inhibits the growth of xenograft tumors derived from Kir2.1-overexpressing non-WNT/SHH MB cells

**Fig. 7:**
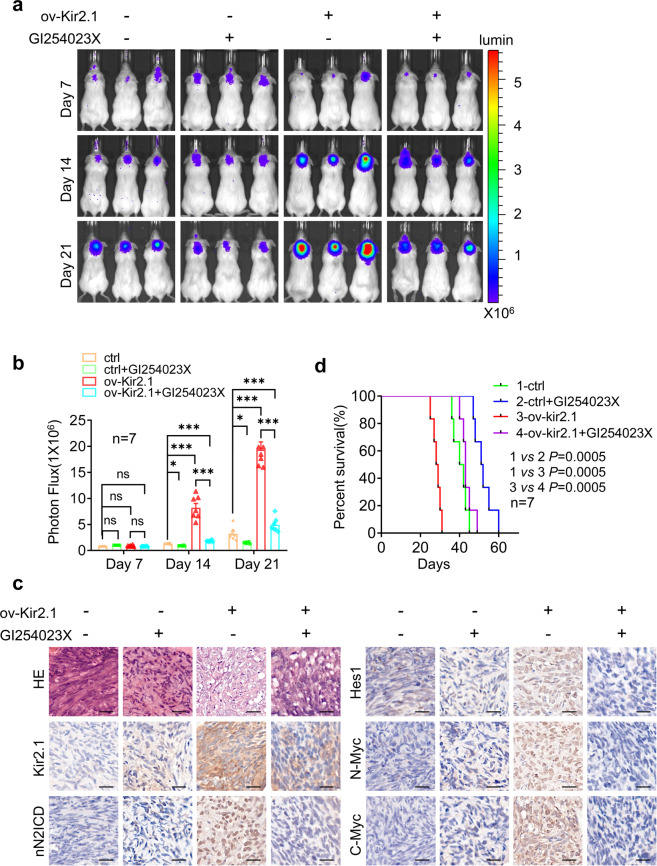
Disruption of Notch2 pathway inhibits the growth and metastasis of Kir2.1- overexpressing MB cell-derived xenograft tumors. **a**, **b** Representative bioluminescent images and the quantification of bioluminescent intensity showing that treatment with Notch2 inhibitor GI254023X (intraperitoneally injected, 100 mg/kg/day, 5 days per week, for 2 weeks) inhibited the growth of xenograft tumors derived from both Kir2.1-overexpressing and control MB428 cells, with more effective inhibition in Kir2.1-overexpressing cell-tumors. On Day 21, GI254023X treatment showed an inhibitory trend on spinal metastasis. Data are shown as mean ± S.D., *n* = 7, ns, not significant, *P < 0.05, ***P < 0.0001, ANOVA test. **c** GI254023X treatment consistently inhibited the nuclear translocation of N2ICD and the expression levels of Hes1, C-Myc, and N-Myc. Scale bar = 20 μm. **d** Kaplan–Meier analysis indicating that prolonged lifespan of GI254023X treated tumor-bearing mice. *n* = 7, *P* = 0.0005, Log-rank test

Since Kir2.1 was associated with the activation of Notch2 signaling in the Kir2.1^high^/nN2ICD^high^ subtype MB, we hypothesized that disrupting the Notch pathway might be a potential therapeutic option. To test the hypothesis, mice bearing orthotopic tumor xenografts derived from Kir2.1-overexpressing MB428 and paired control MB cells were treated with an S2 cleavage inhibitor, GI254023X. GI254023X treatment more potently inhibited the growth of tumors formed by Kir2.1-overexpressing MB cells compared to tumors derived from control MB cells (Fig. 7a, b). Moreover, on Day 21, GI254023X treatment showed an inhibitory trend on spinal metastasis (Fig. 7a). GI254023X treatment consistently inhibited the nuclear translocation of nN2ICD and the expression levels of Hes1, C-Myc, and N-Myc (Fig. 7c). Kaplan–Meier analysis indicated the significantly prolonged lifespan of orthotopic tumor-bearing mice treated with GI254023X as compared to mice without treatment (Fig. 7d). Therefore, inhibitors of the Notch2 signaling pathway are promising therapeutic agents for patients with Kir2.1^high^/nN2ICD^high^ subtype MBs.

## Discussion

Unlike WNT and SHH subgroup MBs, non-WNT/SHH MBs are genetically heterogeneous without specific drivers, key signaling pathways, and mutations in known cancer predisposition genes.^57^ Recently, several studies have divided non-WNT/SHH MBs into different subtypes based on biological and molecular features through various analytical approaches, parameters, and cohorts. It has become a trend that the primary definition of non-WNT/SHH subgroup MBs may be replaced by more specific definitions.^57^ In the present study, we defined a new subtype MBs based on the expression status of Kir2.1/nN2ICD in non-WNT/SHH MBs, in which patients with Kir2.1^high^/nN2ICD^high^ subtype exhibited poorer prognosis than those with non-Kir2.1^high^/nN2ICD^high^ tumors.

Kir2.1 as a member of the inward-rectifier K^+^ channels is involved in cancer progression by either its K^+^ channel function^58^ or its interaction with other molecules.^41^ Our study verifies that the function of Kir2.1 in non-WNT/SHH MB cells is independent of its K^+^ channel function. We also identified Notch2 as an important Kir2.1-interacting protein in MB cells, different from previous findings in GC cells in which Kir2.1 interacts with STK38.^41^ Thus, differential mechanisms are utilized by Kir2.1 to promote the progression of cancer with different origins.

Leptomeningeal dissemination (LMD) is the defining pattern of metastasis for MBs, and spread outside the CNS is rare.^59^ This metastatic pattern led to a proposed LMD cascade of MB cell dispersal through the cerebrospinal fluid (CSF), which is analogous to the invasion-metastasis cascade of hematogenous metastasis of carcinomas. The LMD cascade was envisioned as a 3-stage process: initiation, dispersal, and colonization. In the initiation stage, MB cells escape from the primary tumor mass in the cerebellum and enter the CSF; In the dispersal stage, surviving cells spread through the CSF channels; In the colonization stage, disseminated cells or cell aggregates implant on a distant pial surface and establish a metastatic nidus.^60^ Nevertheless, Garzia et al*.* recently found that MB cells can also spread through the blood to the leptomeningeal space to form leptomeningeal metastases, presenting an alternative hematogenous route for LMD.^61^ However, whether through CSF or hematogenous route, an important prerequisite for the occurrence of LMD is the enhancement of MB cell migration and invasion capabilities. It is well known that EMT plays critical and intricate roles in promoting tumor invasion and metastasis in epithelium-derived carcinomas.^62^ In recent years, many studies have found that the EMT process is also involved in the invasion and metastasis of non-epithelium-derived tumors. For instance, although sarcoma arises from primitively transformed cells with a mesenchymal origin, EMT-related transcription factors such as Twist, Snail, Slug, Zeb1, and Zeb2 play an important role in maintaining the mesenchymal status and the invasion and metastasis abilities of sarcoma cells.^63^ EMT-inducing factors, including the role of Twist1, Zeb1/Zeb2, and Snail1/Snail2, are also presented in malignant glioma (GBM), a brain tumor of neuroepithelium origin, and induce EMT-like changes of GBM cells.^64^ Nevertheless, these typically vary from those observed in epithelial cancers, as no basement membrane is present and the most important cell-cell contact factor, E-cadherin, is rarely expressed in GBM.^65^ A few studies have suggested that the EMT process is involved in MB invasion and metastasis. Gupta et al*.* found that intermittent hypoxia effectively induced EMT phenotypes in MB cells, resulting in significant upregulation of mesenchymal markers Snail, Vimentin, and N-cadherin, and significantly downregulation of epithelial markers ZO-1 and E-cadherin.^66^ Besharat et al*.* reported that there was a EMT molecular network in SHH MB cancer stem cells, which sustained the mesenchymal phenotype of the cells.^67^ In the present study, we demonstrated the involvement of EMT in Kir2.1-promoted invasion and metastasis of non-WNT/SHH MB cells, where manipulating Kir2.1 expression accordingly changed the expression of E-cadherin, Vimentin, and Slug.

Notch signaling pathway determines cell fate in the development. Aberrant activation of the Notch pathway exists in many types of solid tumors, including MB.^68,69^ Recent studies suggest that aberrant Notch signaling in MB is subgroup specific. Genome-wide Cox regression and Gene Set Enrichment Analysis of data from 530 patients revealed that the Notch network is involved in the progression of Group 3 and Group 4 MB.^70^ However, Notch signaling appears not to be involved in SHH MBs.^71^ Notch receptor contains 4 isoforms, i.e., Notch1 – Notch4, which may be differentially involved in the development of MBs. It has been reported that Notch1 expression is directly associated with the metastasis and decreased survival of a mouse MB model, and as a pivotal driver of metastasis and self-renewal of human Group 3 MB cells.^72^ Meanwhile, other research also showed that Notch2 but not Notch1, 3, 4 was overexpressed in MBs.^73^ Our study demonstrates that Notch2 was the main molecule involved in the pathway of Kir2.1 in non-WNT/SHH MBs, emphasizing its critical involvement in the progression of MBs.

Activation of the canonical Notch receptor cascade is initialed by its interaction with the ligands JAG1, JAG2, DLL1, DLL3, and DLL4.^74^ Ligand binding triggers the proteolytic cleavage of Notch by ADAM metalloproteases (S2), a central step for Notch activation, in which the ligands exert a pulling force to alter the structure of the negative regulatory region (NRR) domain in Notch, resulting in Notch cleavage by ADAM metalloproteases.^51^ This process may also occur in Kir2.1-facilitated S2 cleavage of Notch2 by Adam10 as shown in our study, where Kir2.1 appears to form a complex with both Adam10 and Notch2 to trigger a change in NRR structure.

*C-Myc* gene amplification is a defining feature of Group 3 MBs and occurs in ~17% of the tumors.^11^ Patients with *C-Myc*-amplified MBs suffer from an extremely shorter 5-year survival rate of 20%,^75,76^ implying *C-Myc* as an important pro-malignant driver in MBs. Interestingly, *C-Myc* is a gene directly targeted by the Notch2 signaling pathway.^77^ Therefore, upregulation of *C-Myc* by Kir2.1-activated Notch2 signaling might contribute to the malignant phenotype of non-WNT/SHH MBs.

One of the main clinical implications of subclassifying non-WNT/SHH MBs is to innovate the risk stratification and personalize the existing treatment options, as risk-adapted treatment strategies are of paramount importance for the management of MB patients.^70^ Currently, there are different subtypes of non-WNT/SHH MBs, with divergent prognosis. For example, in the I–VIII subtypes of Group 3 and Group 4 MBs,^20,21^ subtypes II and III tumors had a poorer survival rate of 5-year OS 49% and 41%, respectively. In contrast, patients with I, V, VI, VII, and VIII subtype MBs had an 80% 5-year OS; with an even better OS for patients with subtype IV tumors. In our subtyping of non-WNT/SHH MBs, OSs of patients at 500-day after surgery were markedly diverse, with Kir2.1^high^/nN2ICD^high^ subtype at 25% but non-Kir2.1^high^/nN2ICD^high^ subtype at 65%. Despite some differences in reported OSs, our subtype definition in non-WNT/SHH MB should be more suitable for clinical design of therapeutic regimens. Although several candidate drivers have been discovered within subtypes of non-WNT/SHH MBs, such as C-Myc, N-Myc, GFI1B, KBDB4, and KDM6A,^21^ these ‘drivers’ may better be utilized as molecular markers for specific subtypes, rather than as drug targets. By contrast, Kir2.1^high^/nN2ICD^high^ subtype defined in our study harbors specific pathway activation and there are available agents for targeted therapies.

Our findings demonstrate that Kir2.1 is preferentially expressed in non-WNT/SHH MBs and by recruiting Adam10 to enhance the S2 cleavage of Notch2, thereby activating the Notch2 signaling pathway in the MB subgroup. Kir2.1^high^/nN2ICD^high^ MBs harbor the worst outcome and could be defined as a novel subtype in non-WNT/SHH MBs. Our current study would help select daily treatment regimens and develop new targeted therapies for non-WNT/SHH MBs.

## Materials and methods

### Patients and specimens

A total of 170 formalin-fixed and paraffin-embedded surgical MB specimens were collected from patients enrolled in the Southwest Hospital, Army Medical University, and the First Affiliated Hospital of University of Science and Technology of China from 2006 to 2019. All patients did not receive radiotherapy or chemotherapy before surgery. Follow-up information was available for 99 of 170 patients. The tumors were divided into WNT, SHH, and Group 3/4 subgroups according to the 2016 World Health Organization Classification of Tumors of the Central Nervous System.^2^ Written informed consent was obtained from the patients or their guardians. This study was performed by the principles of the Helsinki Declaration and approved by the Ethical Committee of the Southwest Hospital (KY2020220).

### Cells and cell culture

MB428 and MB913 cell lines and MB726 cell line derived from freshly resected non-WNT/SHH and SHH MB tumors respectively were established in our institute (detailed information of MB primary cell lines was listed in Supplementary Table 2). Human Daoy SHH MB cell, ons76 SHH MB cell, D283 Group 3 MB cell, and D341 Group 4 MB cell lines were purchased from American Type Culture Collection (ATCC, USA). All cells were cultured in Dulbecco’s modification of Eagle’s medium (DMEM, Gibico, USA) supplemented with 10% (v/v) fetal bovine serum (FBS, HyClone, USA), 2 mM L-glutamine, 2 mM sodium pyruvate, 100 U/mL penicillin, and 100 µg/mL streptomycin, and maintained in a humidified atmosphere containing 5% CO_2_ at 37 °C.

### Immunohistochemistry

Immunohistochemical (IHC) staining was performed as previously described.^41^ Briefly, after deparaffinization, rehydrated in graded ethanol, antigen retrieval, and blocking, tissue slides were incubated with primary antibodies (detailed information of antibodies was listed in the Supplementary Table 3) at 4 °C overnight. After washing with phosphate-buffered saline (PBS), the corresponding horseradish peroxidase (HRP)-conjugated secondary antibody (DAKO, Denmark) was added and incubated at 37 °C for 30 min. The sections were then stained by 3, 39-diaminobenzidine (DAB, DAKO, Denmark) and counterstained with hematoxylin.

The subgrouping of MB specimens by IHC analyses was performed as previously described.^78^

IHC scoring of Kir2.1 level was performed as previously described.^41^ Briefly, five random IHC images of each slide were captured using an Olympus BX51 microscope (Olympus, Japan). The area sum and integrated optical density (IOD) sum of the positive sites in the images (brown) were measured in pixels by using an Image-Pro Plus 5.0 software. The intensity of Kir2.1 was expressed by the mean value of IOD sum/area sum of 5 photographs for each slide. To ensure data comparability, the same parameter settings were utilized for all photographs. The best cut-off value of the Kir2.1 intensity was determined by the median value of 0.078. The samples with Kir2.1 intensity ≥ 0.078 were defined as high Kir2.1 expression (Kir2.1^high^), relative to low expression of Kir2.1 (Kir2.1^low^).

The IHC staining score of nN2ICD was calculated by multiplying the staining intensity by the percentage of positive cells. The intensity of IHC staining was determined as: 0, negative; 1, weak; 2, moderate; and 3, strong. The percentage of positive cells was scored from 1 (under 25%), 2 (26%–50%), 3 (51%–75%) to 4 (76%–100%). The best predictive cut-off value was determined by the median 8. Cases with scores ≥8 were defined as high nN2ICD expression (nN2ICD^high^), otherwise, they were defined as low expression (nN2ICD^low^).

### Matrigel invasion and wound-healing assays

Matrigel invasion assay was performed using Transwell cell culture chambers (24 wells, 8-μm pore size; Corning, USA). The upper inserts were coated with 10 μL Matrigel (Matrigel: DMEM = 1:3, v/v; BD, USA). Tumor cells (5 × 10^4^) re-suspended in 200 μL serum-free DMEM was added to the upper inserts. DMEM (500 μL) with 10% FBS was added to the lower chambers. After 24 h, invading cells were fixed and stained with crystal violet. Non-invading cells in the upper membrane were removed with a cotton swab. Invading cells were counted in five different fields for each insert at a magnification of ×200 under a light microscope. The experiments were repeated at least three times. For the wound-healing assay, tumor cells were grown to full confluence in 24-well plates, and a straight scratch was then generated manually using a 10 µL pipette tip. The cells were further incubated for 48 h in DMEM without FBS. The widths of the wounds were determined and photographed under light microscopy at 0 and 48 h of wound establishment.

### Whole-cell patch-clamp recording

Potassium current was measured by whole-cell patch-clamp as previously described^41^ at room temperature. Bath (external) solutions were perfused into the chamber using a gravity-driven perfusion system. The standard bath solution consisted of (in mM): 100 D-glucose, 5 KCl, 2 MgCl_2_, 50 NaOH, and 5 glucose. Recording pipettes were filled with an intracellular solution containing (in mM): 110 KCl, 5 Mg ATP, 5 NaCl, 2 MgCl_2_, and 5 glucose. Patch pipettes were made from thin-walled borosilicate glass and fire-polished with a microforge. Data were acquired using an Axopatch 200B amplifier and filtered at 5 Hz with a Digi data 1322A board. Acquisition and analysis were performed using an EPC-10 software.

### RNA extraction and quantitative reverse-transcription PCR (qRT-PCR)

Total RNA was extracted with RNAiso reagent (Takara, Japan). Reverse-transcription and PCR were performed using RNA PCR (AMV) kit (Takara, Japan) with primers designed for human genes. The sequences of each primer pair and the product size were listed in Supplementary Table 4. β-actin was used as a control. The relative gene expression level was calculated using the 2^−^^△△Ct^ method. The cycling conditions were 5 min at 95 °C, 40 cycles of 10 s at 95 °C, 15 s at 60 °C, 20 s at 72 °C, and 5 s at 85 °C. Assays were performed in triplicate.

### Kir2.1 overexpression, knockdown, and site-directed mutagenesis

The lentivirus particles for Kir2.1 overexpression and mutation (144–146: Gly-Tyr-Gly to Ala-Ala-Ala)^40^ were established by Life Technologies Co. Ltd (Shanghai, China). Lentivirus particles for Kir2.1 knockdown and mock were purchased from Santa Cruze (sc-42612-v, sc-108084, USA). All lentivirus particles were used to infect MB cells with 2 µg/mL polybrene and stably transfected cells were selected by 5 µg/mL Puromycin. The efficiency of knockdown and overexpression of Kir2.1 was determined by qRT-PCR and Western blotting.

### Western blotting

MB cells were harvested in cold Radio-Immunoprecipitation Assay (RIPA) buffer (Beyotime Biotech, China) with the protease inhibitor phenylmethanesulfonyl fluoride (PMSF, Beyotime Biotech, China) and incubated on ice for 20 min. After centrifugation at 13,000 rpm at 4 °C for 20 min, the supernatant was collected and measured for protein concentration (BCA protein Assay, Pierce, USA). Equal amount of proteins (20 µg/well) was separated by 10% sodium dodecyl sulfate–polyacrylamide gel electrophoresis (SDS-PAGE) and transferred onto polyvinylidene difluoride (PVDF) membranes (Millipore, USA) at 4 °C. After block with PBST-5% skimmed milk block, the membranes were incubated with primary antibodies (Supplementary Table 3) overnight at 4 °C, then were washed and incubated with corresponding secondary HRP-conjugated antibodies (Beyotime Biotech, China) for 2 h at room temperature. Proteins were visualized with SuperSignal West Femto Maximum Sensitivity Substrate (ECL, Thermo Fisher Scientific, USA) and detected by a ChemiDocXRS system (Bio-Rad, USA).

### Mouse orthotopic models and drug treatments

Mice were purchased from the Laboratory Animal Center, Army Medical University (Chongqing, China). To establish mouse orthotopic models, MB428 and MB913 cells were injected into the cerebellum of 6-week-old NOD-SCID mice at 1 × 10^5^ cells per mouse (*n* = 7). The tumorigenesis and metastasis were monitored by bioluminescent imaging on Day 14, Day 28, and Day 42 using In Vivo Imaging System (IVIS) Spectrum (Perkin-Elmer, USA). The mouse survival was observed for up to all the animals dead. NOD-SCID mouse models established with MB428 cells as above were used for drug studies. On the 7th day after implantation, animals were intraperitoneally injected with Adam10 inhibitor, GI254023X (Selleck; formulated in 10% DMSO in 0.1 M carbonate buffer), 100 mg/kg/day, 5 days per week, for two weeks. Control groups were given identical volume of vehicle instead of GI254023X. Xenograft growth and metastasis were monitored by bioluminescent imaging using In Vivo Imaging System (IVIS) Spectrum (Perkin-Elmer, USA) at 7, 14, and 21 days. The animal experiments were approved by the Institutional Animal Care and Use Committee of Army Medical University (AMUWEC20201439).

### Immunoprecipitation-mass spectrometry and co-immunoprecipitation

Immunoprecipitation (IP) was performed using a IP kit (#26149, Thermo Scientific, USA) following the manufacturer’s protocol. Briefly, 10 µg Flag antibody and IgG were immobilized on AminoLink Plus coupling resin for 2 h, respectively. 250 µg MB cell lysate was added and incubated at 4 °C overnight. Then the resin was washed and eluted using an elution buffer. The eluted proteins were sent to The Central Laboratory of the Army Medical University for mass spectrometry. For Co-IP experiment, the eluted proteins were separated by SDS-PAGE and immunoblotted with indicated antibodies (detailed information was listed in Supplementary Table 3).

### Confocal laser scanning microscopy

Kir2.1-overexpressing and control MB428 cells (1 × 10^4^/well) were cultured in a 4-well Lab-Tak chamber for 2 days. The cells were washed three times with a growth medium and fixed overnight using 4% paraformaldehyde. After permeabilizing with 0.2% Triton X-100 for 10 min at room temperature (RT), the cells were blocked with 4% BSA in PBS at 4°C for 1 h. Then a mouse anti-human Kir2.1 antibody (ab109750, Abcam, UK) or a rabbit anti-human N2ICD antibody (HPA048743, Atlas Antibodies, Sweden) was added and incubated at 4 °C overnight. After washing three times with PBS, the cells were incubated with Alexa 488 labeled donkey anti-mouse IgG (H + L) and Alexa 555 labeled goat anti-rabbit IgG (H + L) (Invitrogen, USA) at 37 °C for 30 min. Cell nuclei were then stained with 4,6-diamino-2-phenyl indole (DAPI). All samples were then analyzed by a confocal laser scanning microscopy (SP-5, Leica, Germany).

### FACS analysis and cell sorting

Kir2.1-overexpressing and control MB single cells under different treatments were labeled with APC-conjugated anti-human Notch2 antibody (130-096-972, MACS, Germany) for 30 min at RT with APC-conjugated IgG (130-093-197, MACS, Germany) as control. The levels of Notch2 were detected by the FACSAria II cell sorter (BD, USA).

### Nuclear and cytoplasmic protein extraction

Nuclear protein extraction was performed by using a NE-PER Nuclear and Cytoplasmic Extraction Reagents kit (#78833, Thermo Scientific, USA) following the manufacturer’s protocol. Briefly, added ice-cold CER I to the cell pellet, then vortexed the tube vigorously on the highest setting. Added ice-cold CER II to the tube and centrifuged the tube and transferred the supernatant (cytoplasmic extract) to a clean pre-chilled tube. Then suspended the insoluble (pellet) fraction containing nuclei with ice-cold NER. After centrifuging, the supernatant (nuclear extract) fraction was immediately transferred to a clean pre-chilled tube. The extracted nuclear and cytoplasmic proteins were used for subsequent experiments.

### Membrane protein extraction

Membrane Protein Extraction was performed by using a Mem-PER^TM^ Plus Membrane Protein Extraction Kit (#89842, Thermo Scientific, USA) following the manufacturer’s protocol. Briefly, washed cell pellet with Cell Wash Solution, then added Permeabilization Buffer to the cell pellet. Centrifuged permeabilized cells and removed the supernatant containing cytosolic proteins. Added Solubilization Buffer to re-suspend by pipetting up and down. After centrifuging, transferred supernatant containing solubilized membrane proteins to a new tube. The extracted membrane protein was for subsequent experiments.

### Statistics

All experiments were conducted at least three times. Results were presented as the mean ± standard deviation (SD). The statistical significance between testing and control groups was analyzed with SPSS20.0 statistical software and GraphPad Prism. Chi-square analysis was used to evaluate the relationship between Kir2.1^high^ rate and subgroup of medulloblastoma. The significance was determined by a two-tailed unpaired Student’s *t*-test or one-way ANOVA. Kaplan–Meier curves and log-rank analysis were used to measure survival. Cox’s proportional hazard regression model was established for multivariate analysis of the combined contribution of Kir2.1/nN2ICD status and clinicopathological features to the OS of patients. A *P* value < 0.05 was considered statistically significant.

## Supplementary information

SUPPLEMENTAL MATERIAL

## Data Availability

All data generated or analyzed during this study are included in this manuscript and its supplementary information files. All data from GSE28245 and GSE37418 is available at its website: https://www.ncbi.nlm.nih.gov/geo/.
