# Supplementary material for: Elevated Kir2.1/nuclear N2ICD defines a highly malignant subtype of non-WNT/SHH medulloblastomas
Source: Signal Transduct Target Ther. 2022 Mar 11;7:72. doi: 10.1038/s41392-022-00890-7 (PMC8913686; doi:10.1038/s41392-022-00890-7)
Supplement: Supplementary file 1 — SUPPLEMENTAL MATERIAL [file 41392_2022_890_MOESM1_ESM.docx]

Supplementary Materials for

**Elevated Kir2.1/nuclear Notch2 Defines a Highly Malignant Subtype of non-WNT/SHH Medulloblastomas**

Paste Yan-Xia Wang^1#^, Haibo Wu^2,3#^, Yong Ren^4^, Shengqing Lv^5^, Chengdong Ji^1^, Dongfang Xiang^1^, Mengsi Zhang^1^, Huimin Lu^1^, Wenjuan Fu^1^, Qing Liu^1^, Zexuan Yan^1^, Qinghua Ma^1^, Jingya Miao^1^, Ruili Cai^1^, Xi Lan^1^, Bin Wu^1^, Wenying Wang^1^, Yinhua Liu^6^, Dai-Zhong Wang^7^, Mianfu Cao^1^, Zhicheng He^1^, Yu Shi^1^, Yifang Ping^1^, Xiaohong Yao^1^, Xia Zhang^1^, Peng Zhang^1^, Ji Ming Wang^8^, Yan Wang^1^*, Youhong Cui^1^*, and Xiu-Wu Bian^1^*

Correspondence to: bianxiuwu@263.net; cuiyouhongx@yahoo.com; wang_yan1977@hotmail.com

**This file includes:**

**Figures. S1 to S15**

**Tables S1 to S5**

Figure. S1.

**
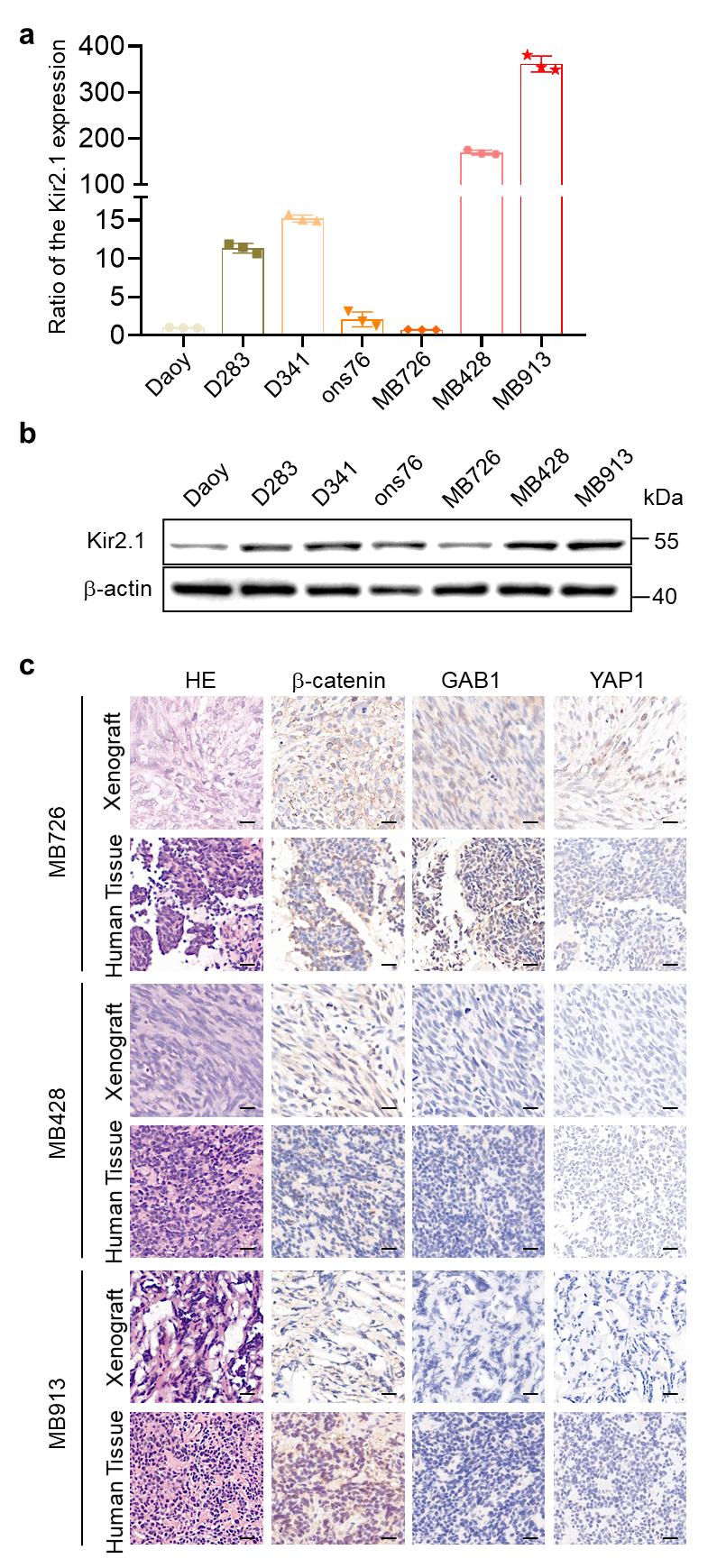
**

**Supplementary Fig. 1 Detection of Kir2.1 expression in MB cell lines and primary MB cells.**

**a** Higher mRNA levels of Kir2.1 in non-WNT/SHH MB cells (D283, D341, MB428 and MB913) than in SHH subgroup MB cell lines (Daoy, ons76 and MB726) detected by qRT-PCR. Data are shown as mean ± S.D., n=3. **b** Higher protein levels of Kir2.1 in non-WNT/SHH MB cells than in SHH subgroup MB cell lines detected by Western blotting. **c** The subgroup of the primary cell lines MB726, MB428 and MB913 was validated by IHC staining on the sections of xenograft tumors derived from those cells. The human tissue is the original surgically removed sample from the same fresh sample that was used to establish the primary cell line. Scale bar = 20 μm.

Figure. S2.


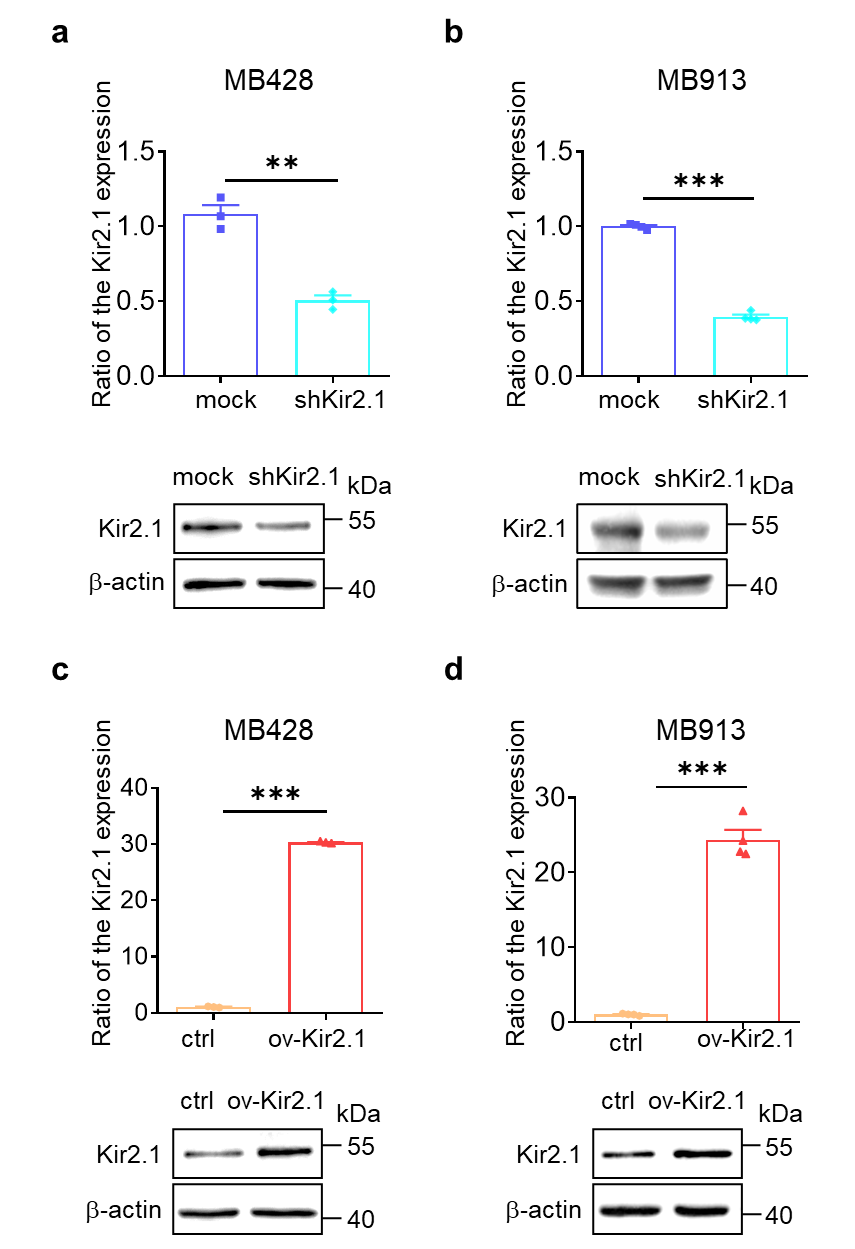


**Supplementary Fig. 2 The efficacy of Kir2.1 knockdown and overexpression in MB cells.**

**a, b** The efficacy of Kir2.1 knockdown (Santa Cruze, sc-42612-v and sc-108084 (mock)) in MB428 and MB913 cells at mRNA and protein levels measured by qRT-PCR and Western blotting, respectively. Data are shown as mean ± S.D., n=3, **, *P* < 0.001, ***, *P* < 0.0001, Student’s t-test. **c,** **d** The efficacy of Kir2.1 overexpression in MB428 and MB913 cells at mRNA and protein levels measured by qRT-PCR and Western blotting, respectively. Data are shown as mean ± S.D., n=3, **, *P* < 0.001, ***, *P* < 0.0001, Student’s t-test.

Figure. S3.


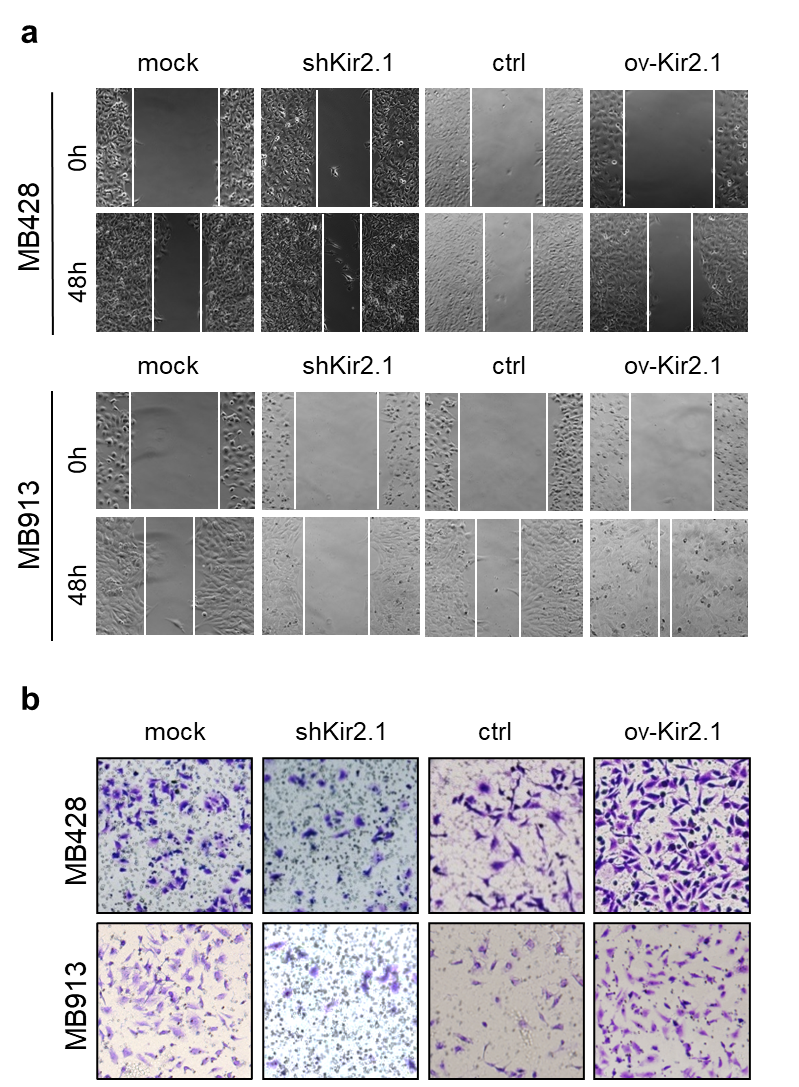


**Supplementary Fig. 3 Representative images of migration and invasion affected by Kir2.1-knockdown and -overexpression in MB cells. a** Representative images of wound-healing assay showing that Kir2.1-knockdown markedly reduced MB428 and MB913 cell migration into the scratching area, while MB cells with Kir2.1 overexpression migrated more rapidly into the scratching area than control cells. **b** Representative images of matrigel-transwell invasion assay showing decreased invasive capability of Kir2.1-knockdown MB428 and MB913 cells, and the opposite results in Kir2.1-overexpressing MB cells.

Figure. S4.


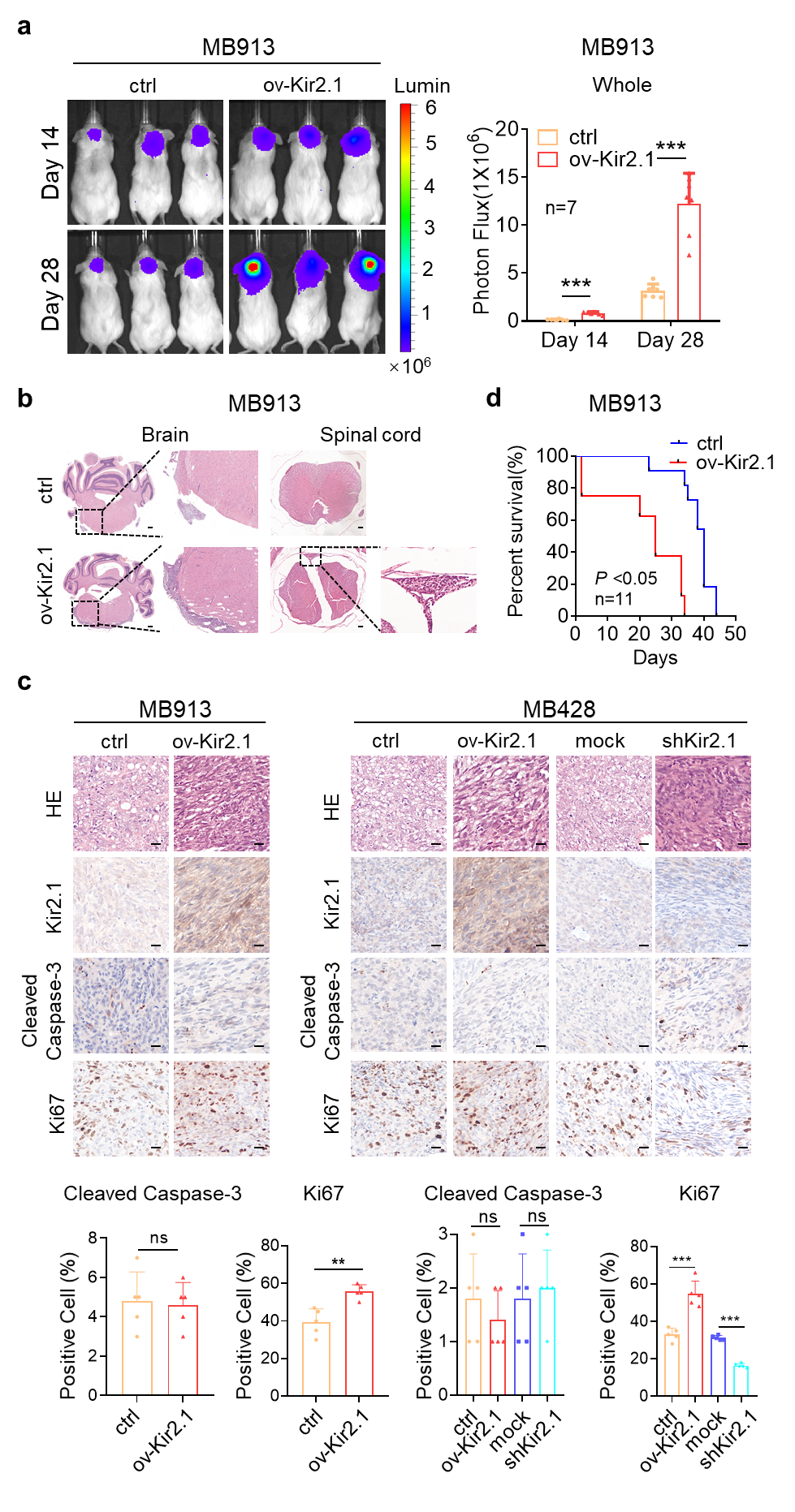


**Supplementary Fig. 4 Overexpressing Kir2.1 increases the metastatic capacities of MB913 cells *in vivo*.** **a** Representative bioluminescent images (left panel) and the quantiﬁcation (right panel) showing higher tumorigenicity ability of Kir2.1-overexpressing MB913 cells than control cells. Data are shown as mean ± S.D., n=7, ***, *P* < 0.0001, Student’s t-test. **b** Representative HE staining of brain and spinal area sections showing more and larger metastatic foci of Kir2.1-overexpressing MB913 cells than control cells. Scale bar = 100 μm. **c** IHC staining confirmed that the overexpression and knockdown of Kir2.1 in MB cells were stable *in vivo*. Overexpression/silencing of Kir2.1 significantly increased/decreased the percentage of Ki67 positive cells, respectively, but had no significant effect on the percentage of Cleaved Caspase-3 positive cells as compared to the controls. Scale bar = 25 μm. Data are shown as mean ± S.D., n=5, ns, not significant, **, *P* < 0.001, ***, *P* < 0.0001, Student’s t-test. **d** Kaplan–Meier survival curves showing significantly shorter lifespan of the mice bearing xenograft tumors derived from Kir2.1 overexpressing MB913 cells as compared to controls. n=11, *P*＜0.05, Log-rank test.

Figure. S5.


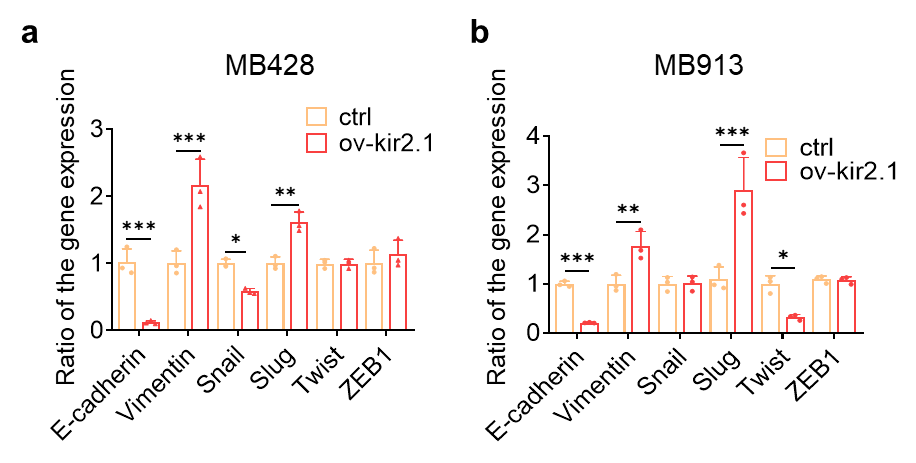


**Supplementary Fig. 5 Detection of major EMT-associated transcription factors affected by Kir2.1 overexpression in non-WNT/SHH MB cells.** **a** qRT-PCR assay showing that overexpressing of Kir2.1 resulted in down-regulation of E-cadherin but up-regulation of Vimentin and Slug in MB428 cells. Data were shown as the mean ± S.D., n = 3, *, *P* < 0. 05; **, *P* < 0.001; ***, *P* < 0.0001, Student’s t-test. **b** qRT-PCR showing that overexpressing of Kir2.1 resulted in down-regulation of E-cadherin but up-regulation of Vimentin and Slug in MB913. Data were shown as the mean ± S.D., n = 3, *, *P* < 0. 05; **, *P* < 0.001; ***, *P* < 0.0001, Student’s t-test.

Figure. S6.


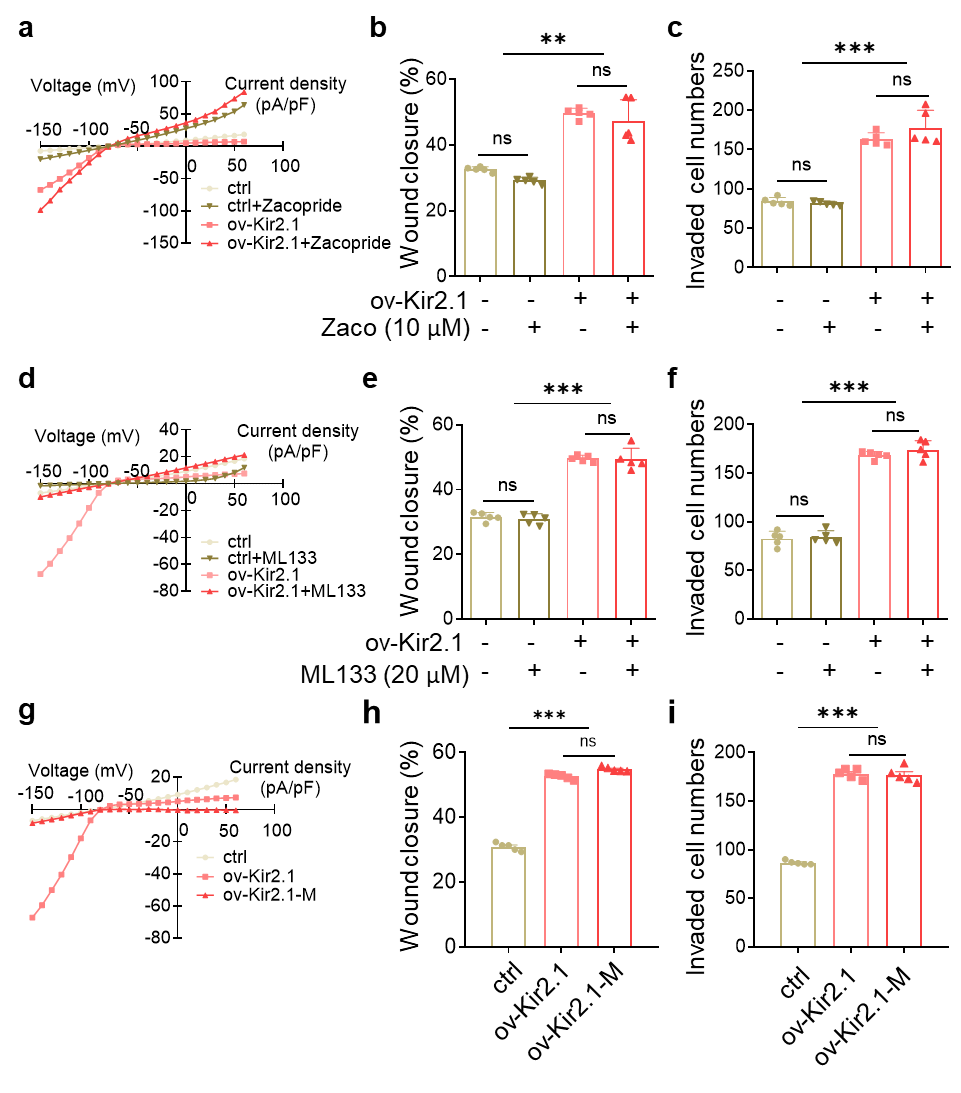


**Supplementary Fig. 6 Kir2.1 promotes the invasion of MB428 cells independent of its K^+^ channel function.** **a** Treatment with the Kir2.1 agonist, Zacopride (10 μM) increased IK^+^ in Kir2.1-expressing MB428 and control cells. **b, c** Treatment with Zacopride (10 μM) did not affect the migratory and invasive capabilities of Kir2.1-expressing MB428 and control cells. Data were shown as the mean ± S.D., n = 5. ns, not significant; **, *P* < 0.001; ***, *P* < 0.0001, ANOVA test. **d** Treatment with ML133 (20 μM), an inhibitor of Kir2.1, abolished inward rectifier potassium current (IK1) in both Kir2.1-overexpressing and control MB428 cells. **e, f** ML133 treatment (20 μM) did not alter the migratory and invasive capabilities of both Kir2.1-overexpressing and control MB428 cells. Data were shown as the mean ± S.D., n = 5. ns, not significant; ***, *P* < 0.0001, ANOVA test. **g** Over-expressing mutant Kir2.1 did not increase IK1 in MB428 cells. **h, i** Over-expressing mutant Kir2.1 significantly elevated the migratory and invasive capabilities of MB428 cells. Data were shown as the mean ± S.D., n = 5. ns, not significant; ***, *P* < 0.0001, ANOVA test.

Figure. S7.


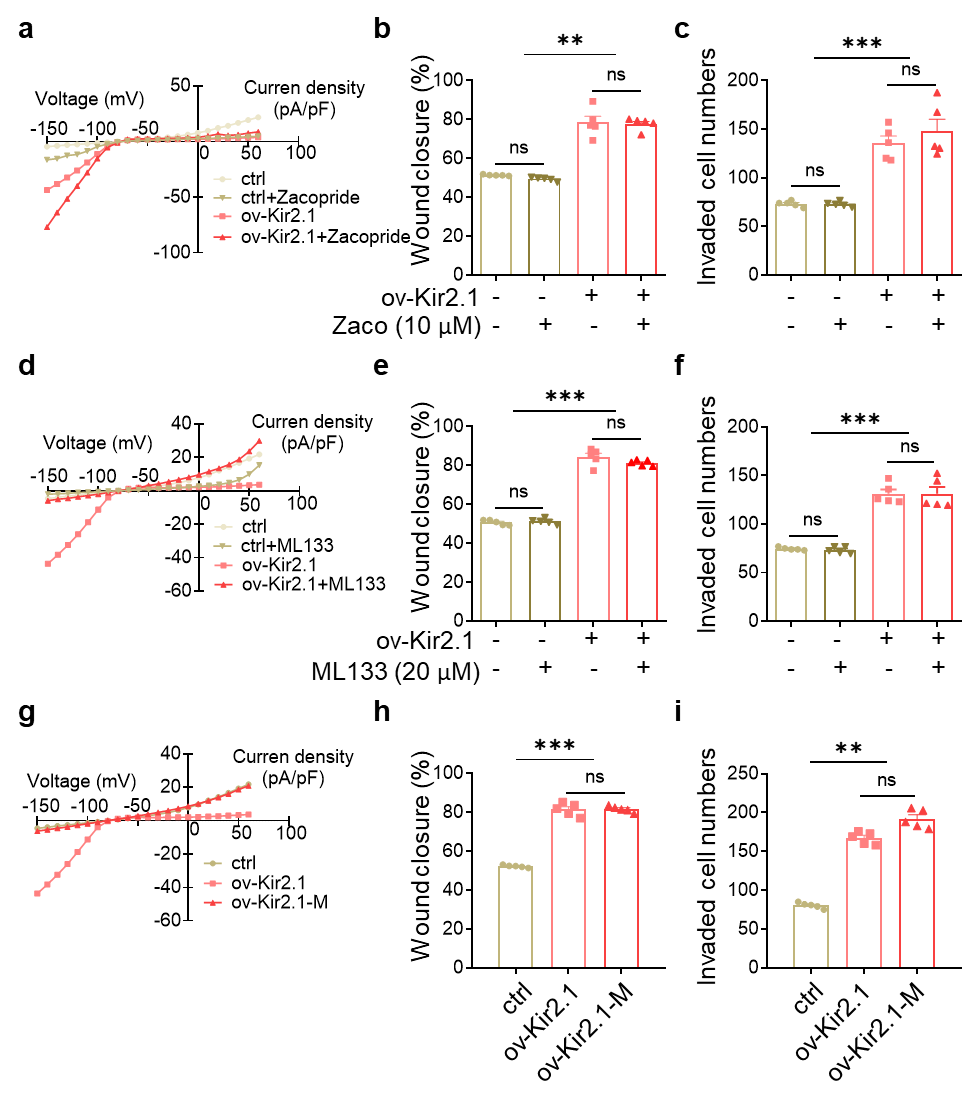


**Supplementary Fig. 7 Kir2.1 promotes the invasiveness of MB913 cells independent of its channel function. a** Treatment with the Kir2.1 agonist, Zacopride (10 μM), increased IK+ in Kir2.1-expressing MB913 and control cells. **b, c** Treatment with Zacopride (10 μM) did not affect the migratory and invasive capabilities in Kir2.1-expressing MB913 and control cells. Data were shown as the mean ± S.D., n = 5. ns, not significant; **, *P* < 0.001; ***, *P* < 0.0001, ANOVA test. **d** Treatment with ML133 (20 μM), an inhibitor of Kir2.1, disrupted IK+ in both Kir2.1-overexpressing and control MB913 cells. **e, f** ML133 (20 μM) treatment did not alter the migratory and invasive capabilities of both Kir2.1-overexpressing and control MB913 cells. Data were shown as the mean ± S.D., n = 5. ns, not significant; ***, *P* < 0.0001, ANOVA test. **g** Over-expressing mutant Kir2.1 in MB913 cells did not increase IK+. **h, i** Over-expressing mutant Kir2.1 markedly elevated the migratory and invasive capabilities of MB913 cells. Data were shown as the mean ± S.D., n = 5. ns, not significant; **, *P* < 0.001; ***, *P* < 0.0001, ANOVA test.

Figure. S8.


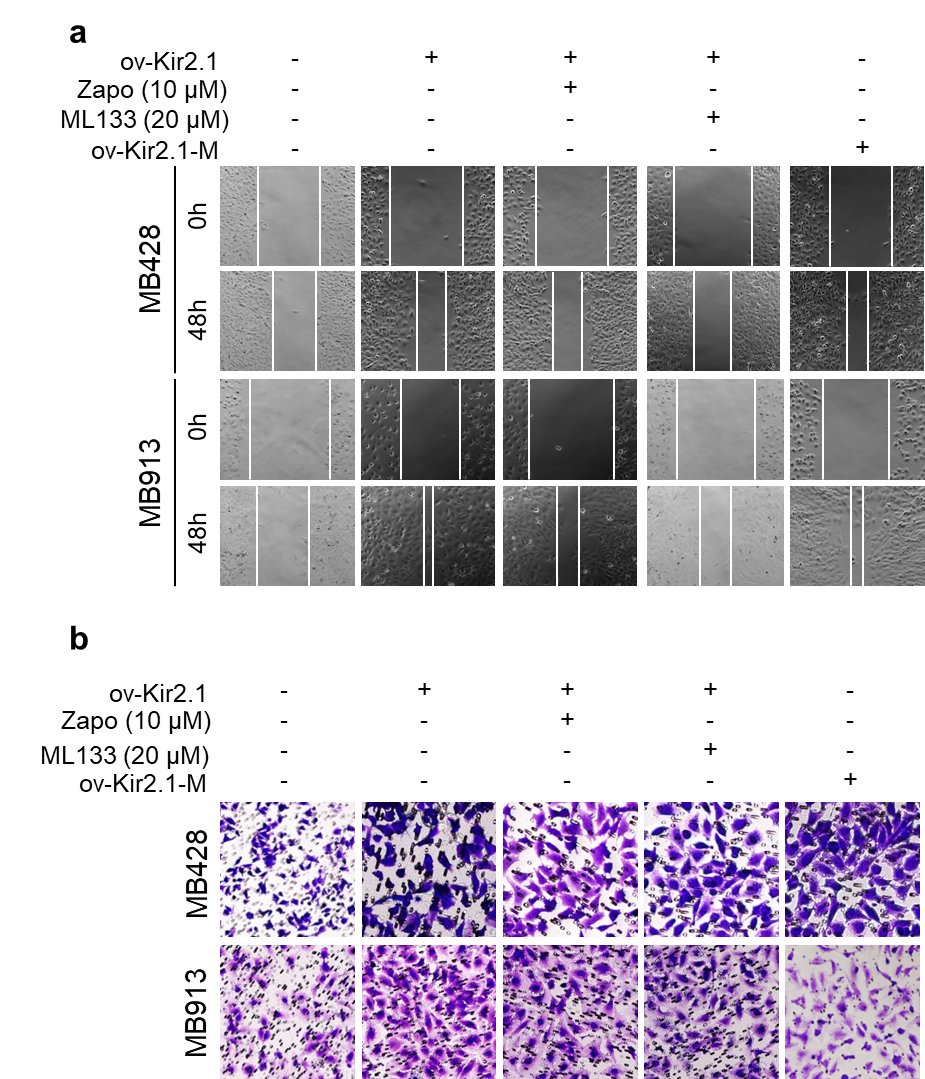


**Supplementary Fig. 8 Representative images of MB cell migration and invasion affected by treatment with stimulator and inhibitor or mutation of Kir2.1.**

**a** Treatment with stimulator and inhibitor or mutation of Kir2.1 did not affect the migratory capability of MB cells. **b** Treatment with stimulator and inhibitor or mutation of Kir2.1 did not affect the invasive capability of MB cells.

Figure. S9.


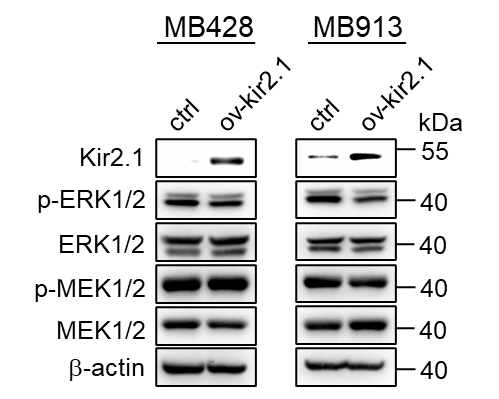


**Supplementary Fig. 9 Kir2.1 does not activate MEK1/2-ERK1/2 pathway in MB cells.**

Western blotting showing that overexpressing Kir2.1 did not alter the phosphorylation level of ERK1/2 and MEK1/2 in MB428 and MB913 cells.

Figure. S10.


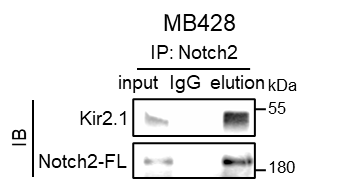


**Supplementary Fig. 10 The interaction between Notch2 and Kir2.1 by Co-IP in wild type MB428.** Co-IP showing that Notch2 interacts with endogenous Kir2.1 in wild type MB428 cells.

Figure. S11.


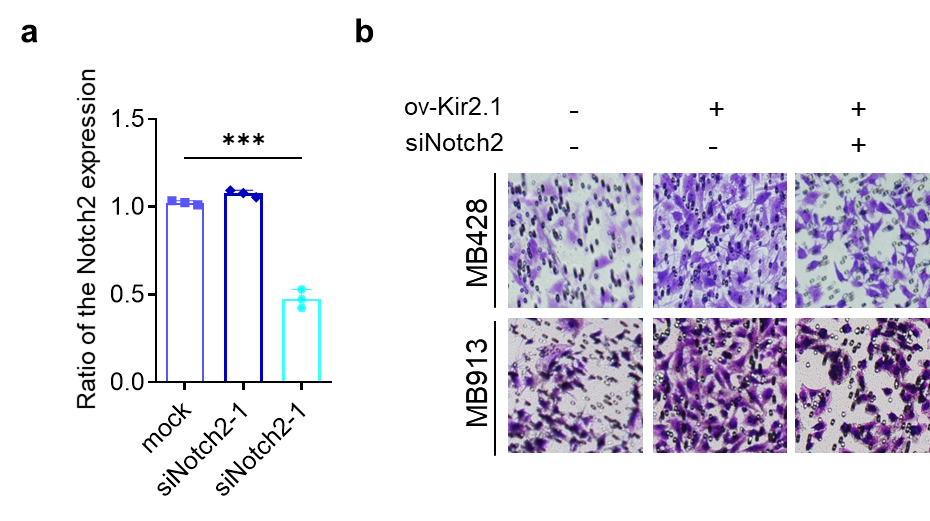


**Supplementary Fig.11 Representative images of MB cell invasion affected by siNotch2.**

**a** The efficiency of Notch2 knockdown in MB cells at mRNA level. **b** Treatment with siNotch2 reduced the invasive capabilities of MB cells. Data are shown as mean ± S.D., n=3, ***, *P* < 0.0001, Student’s t-test.

Figure. S12.


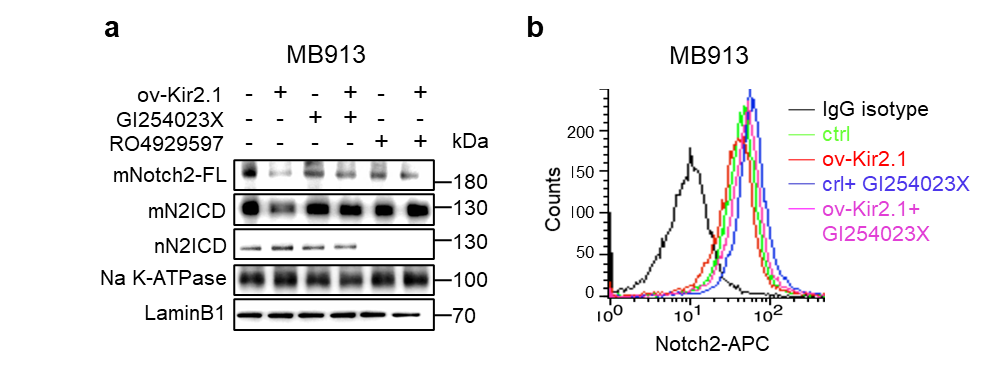


**Supplementary Fig. 12 Kir2.1 enhances S2 cleavage of Notch2 in MB913 cells. a** Treatment with S2 inhibitor GI254023X (3 μM) increased mNotch2-FL and mN2ICD in control cells but abolished the effect of Kir2.1 in Kir2.1-overexpressing MB913 cells detected by Western blotting. Treatment with the S3 inhibitor RO4929597 (10 μM) increased mN2ICD and abolished nN2ICD in both Kir2.1-overexpressing MB913 cells and control cells. **b** FACS showing that S2 cleavage inhibitor attenuated the ability of overexpressing Kir2.1 to reduce the extracellular domain of Notch2 in MB913 cells.

Figure. S13.


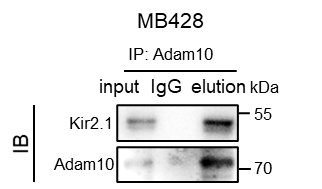


**Supplementary Fig. 13 The interaction between Adam10 and Kir2.1 by Co-IP in wild type MB428.** Co-IP showing that Adam10 interacts with endogenous Kir2.1 in wild type MB428 cells.

Figure. S14.


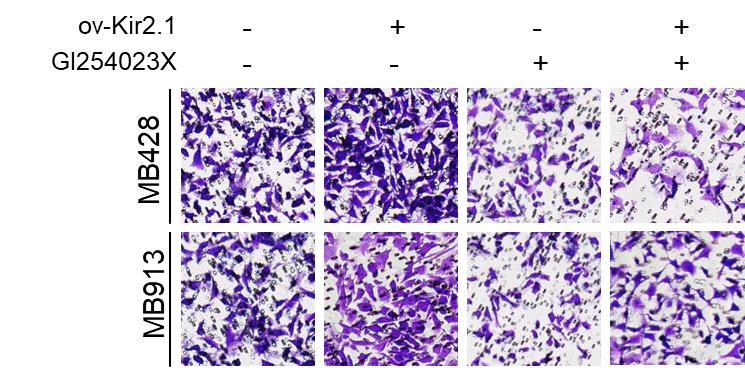


**Supplementary Fig. 14 Representative images of MB cell invasion reduced by the GI254023X.** Treatment with S2 inhibitor GI254023X (3 μM) reduced the invasive capabilities of MB cells.

Figure. S15.


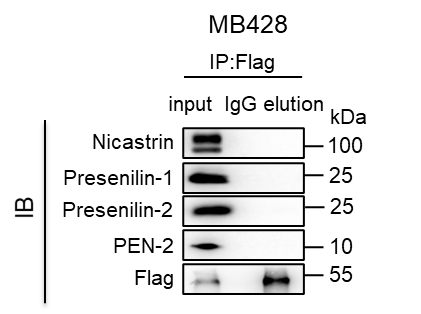


**Supplementary Fig. 15 The interaction between Kir2.1 and γ-Secretase Complex by Co-IP in MB428.** IP showing that Kir2.1 did not physically interact with any component of γ-Secretase Complex, including Nicastrin, Presenilin-1, Presenilin-2 and PEN-2 in Kir2.1-overexpressing (Flag-Kir2.1) MB428 cells.

Table S1.

**Supplementary Table 1** The information of GEO datasets used in this study

|  | **GSE37418** | **GSE28245** |
| --- | --- | --- |
| Website | https://www.ncbi.nlm.nih.gov/geo/query/acc.cgi?acc=GSE37418 | https://www.ncbi.nlm.nih.gov/geo/query/acc.cgi?acc=GSE28245 |
| Organism | Homo sapiens | Homo sapiens |
| Country | USA | Canada |
| Platform | GPL570 | GPL6480 |
| Patient population | 76 patients | 64 patients |
| Data processing | DAT files were analysed by MAS 5.0 to generate background-normalized image data (CEL files). The full data set was normalized according to the global normalization method. | The raw probe intensities were normalized using the quartile normalization approach, and the PLIER method was utilized to summarize the probe signal to both the exon- and gene-level expressions. |
| Age, Sex, Stage, Grade | https://www.ncbi.nlm.nih.gov/geo/query/acc.cgi?acc=GSE37418 | https://www.ncbi.nlm.nih.gov/geo/query/acc.cgi?acc=GSE28245 |

Table S2.

**Supplementary Table 2** The information of MB primary cell lines

| **Patients** | **Age, year** | **Sex** | **Location** | **Pathological subtype** | **Molecular subgroups** |
| --- | --- | --- | --- | --- | --- |
| MB913 | 10 | male | The forth ventricle | classic | non-WNT/SHH |
| MB428 | 15 | female | Cerebellar vermis | classic | non-WNT/SHH |
| MB726 | 6 | male | Cerebellar vermis | desmoplastic | SHH |

Table S3.

**Supplementary Table 3** The information of antibodies used in immunohistochemical and Western blotting experiments

| **Antibodies** | **Source** | **Catalog numbe**r |
| --- | --- | --- |
| Anti-β-catenin (CTNNB1) (D10A8) | Cell Signaling Technology | # 8480 |
| Anti-GAB1(C32H2) | Cell Signaling Technology | #3232 |
| Anti-Kir2.1 [EPR4530] | Abcam | ab109750 |
| Anti-Kir2.1(NGVPESTSTDTPPDIDLHN) | Sigma | P6102 |
| Anti-N2ICD (D76A6) | Cell Signaling Technology | #5732 |
| Anti-E-cadherin (24E10) | Cell Signaling Technology | #3195 |
| Anti-Vimentin (D21H3) | Cell Signaling Technology | #5741 |
| Anti-Slug (C19G7) | Cell Signaling Technology | #9585 |
| Anti-C-Myc (D84C12) | Cell Signaling Technology | #5605 |
| Anti-Hes1 (D6P2U) | Cell Signaling Technology | #11988 |
| Anti-LaminB1 (A-11) | Santa Cruze | Sc-56145 |
| Anti-Na/K ATPase (D4Y7E) | Cell Signaling Technology | #23565 |
| Anti-Actin (8H10D10) | Cell Signaling Technology | #3700 |
| Anti-GAPDH (D16H11) | Cell Signaling Technology | #5174 |
| Anti-Flag (9A3) | Cell Signaling Technology | #8146 |
| Anti-Notch1-FL (D1E11) | Cell Signaling Technology | #3608 |
| Anti-Notch2-FL (8A1) | Cell Signaling Technology | #2420 |
| Anti-Notch3 (D11B8) | Cell Signaling Technology | #5276 |
| Anti-Notch4-FL (L5C5) | Cell Signaling Technology | #2423 |
| Anti-HMGB1 (D3E5) | Cell Signaling Technology | #6893 |
| Anti-HDGF(E3P7K) | Cell Signaling Technology | #42105 |
| Anti-RALA (D6G8) | Cell Signaling Technology | #4799 |
| Anti-TBX2 | Invitrogen | PA5-99727 |
| Anti-Adam10 | Cell Signaling Technology | #14194 |
| Anti-Nicastrin (D4F6N) | Cell Signaling Technology | #[30239](https://www.cellsignal.cn/products/primary-antibodies/nicastrin-d4f6n-rabbit-mab/30239?site-search-type=Products) |
| Anti-Presenilin 1 (D39D1) | Cell Signaling Technology | #5643 |
| Anti-Presenilin 2 (D30G3) | Cell Signaling Technology | #9979 |
| Anti-PEN-2 [(D6G8)](https://www.cellsignal.cn/products/primary-antibodies/8598) | Cell Signaling Technology | #[8598](https://www.cellsignal.cn/products/primary-antibodies/8598) |
| Anti-ERK1/2 (137F5) | Cell Signaling Technology | #4695 |
| Anti-p-ERK1/2 (D13.14.4E) | Cell Signaling Technology | #4370 |
| Anti-MEK1/2 | Cell Signaling Technology | #9122 |
| Anti-p-MEK1/2 (41G9) | Cell Signaling Technology | #9154 |
| Anti-rabbit IgG, HRP-linked Antibody | Cell Signaling Technology | #7074 |
| Anti-mouse IgG, HRP-linked Antibody | Cell Signaling Technology | #7076 |
| Anti-Ki67 | ZSJQB Co., Ltd. | ZM-0166 |
| Anti-Cleaved Caspase-3 (5A1E) | Cell Signaling Technology | #9664 |
| Anti-Yap1 | Abcam | ab205270 |
| Anti-C-Myc | Abcam | ab185656 |
| Anti-N-Myc | Abcam | ab198912 |

Table S4.

**Supplementary Table 4** The primer sequences used in this study

| **Genes** | **Primers sequences** | **Product size** |
| --- | --- | --- |
| Kir2.1 | F:GTGCGAACCAACCGCTACA/R:CCAGCGAATGTCCACACAC | 234 |
| E-cadherin | F:AAAGGCCCATTTCCTAAAAACCT/R:TGCGTTCTCTATCCAGAGGCTG | 172 |
| Vimentin | F:GACGCCATCAACACCGAGTT/R:CTTTGTCGTTGGTTAGCTGGT | 238 |
| Snail | F:ACTGCAACAAGGAATACCTCAG/R:GCACTGGTACTTCTTGACATCTG | 242 |
| Slug | F:TGTGACAAGGAATATGTGAGCC/R:TGAGCCCTCAGATTTGACCTGGGT | 203 |
| Twist | F:GTCCGCAGTCTTACGAGGAG/R:GCTTGAGGGTCTGAATCTTGCT | 156 |
| ZEB1 | F:CAGCTTGATACCTGTGAATGGG/R:TATCTGTGGTCGTGTGGGACT | 185 |
| β-actin | F:CATGTACGTTGCTATCCAGGC/R:CTCCTTAATGTCACGCACGAT | 147 |

Table S5.

**Supplementary Table 5** 77 Potential interactive proteins with Kir2.1

| number | Accession | Description | Score | Coverage | # Proteins |
| --- | --- | --- | --- | --- | --- |
| 1 | P35579 | Myosin-9 OS=Homo sapiens GN=MYH9 PE=1 SV=4 - [MYH9_HUMAN] | 219.05 | 39.34 | 22 |
| 2 | P07355 | Annexin A2 OS=Homo sapiens GN=ANXA2 PE=1 SV=2 - [ANXA2_HUMAN] | 124.51 | 68.14 | 25 |
| 3 | P14618 | Pyruvate kinase PKM OS=Homo sapiens GN=PKM PE=1 SV=4 - [KPYM_HUMAN] | 93.30 | 57.06 | 14 |
| 4 | P52732 | Kinesin-like protein KIF11 OS=Homo sapiens GN=KIF11 PE=1 SV=2 - [KIF11_HUMAN] | 47.11 | 21.21 | 1 |
| 5 | P04083 | Annexin A1 OS=Homo sapiens GN=ANXA1 PE=1 SV=2 - [ANXA1_HUMAN] | 39.50 | 46.24 | 3 |
| 6 | J3KNT0 | Fascin OS=Homo sapiens GN=FSCN1 PE=3 SV=1 - [J3KNT0_HUMAN] | 36.63 | 33.47 | 2 |
| 7 | P23528 | Cofilin-1 OS=Homo sapiens GN=CFL1 PE=1 SV=3 - [COF1_HUMAN] | 30.70 | 63.25 | 9 |
| 8 | G3V1V0 | Myosin light polypeptide 6 OS=Homo sapiens GN=MYL6 PE=2 SV=1 - [G3V1V0_HUMAN] | 28.39 | 57.76 | 17 |
| 9 | P63252 | Inward rectifier potassium channel 2 OS=Homo sapiens GN=KCNJ2 PE=1 SV=1 - [IRK2_HUMAN] | 28.32 | 29.27 | 8 |
| 10 | P54253 | Ataxin-1 OS=Homo sapiens GN=ATXN1 PE=1 SV=2 - [ATX1_HUMAN] | 27.03 | 20.98 | 1 |
| 11 | Q99466 | Notch 2 intracellular domain OS=Homo sapiens GN=NOTCH2 PE=2 SV=1 - [NOTCH2_HUMAN] | 25.19 | 20.07 | 5 |
| 12 | Q16643 | Drebrin OS=Homo sapiens GN=DBN1 PE=1 SV=4 - [DREB_HUMAN] | 23.20 | 9.45 | 8 |
| 13 | A8K8G0 | Hepatoma-derived growth factor OS=Homo sapiens GN=HDGF PE=2 SV=1 - [A8K8G0_HUMAN] | 21.87 | 20.63 | 8 |
| 14 | H7C3P7 | Ras-related protein Ral-A (Fragment) OS=Homo sapiens GN=RALA PE=4 SV=1 - [H7C3P7_HUMAN] | 20.60 | 23.23 | 4 |
| 15 | P55081 | Microfibrillar-associated protein 1 OS=Homo sapiens GN=MFAP1 PE=1 SV=2 - [MFAP1_HUMAN] | 20.28 | 45.74 | 1 |
| 16 | Q5T7C4 | High mobility group protein B1 OS=Homo sapiens GN=HMGB1 PE=2 SV=1 - [Q5T7C4_HUMAN] | 19.32 | 9.38 | 6 |
| 17 | P32119 | Peroxiredoxin-2 OS=Homo sapiens GN=PRDX2 PE=1 SV=5 - [PRDX2_HUMAN] | 19.27 | 14.69 | 2 |
| 18 | P07737 | Profilin-1 OS=Homo sapiens GN=PFN1 PE=1 SV=2 - [PROF1_HUMAN] | 18.74 | 41.95 | 3 |
| 19 | Q13207 | T-box transcription factor TBX2 OS=Homo sapiens GN=TBX2 PE=1 SV=3 - [TBX2_HUMAN] | 18.58 | 57.79 | 12 |
| 20 | P14923 | Junction plakoglobin OS=Homo sapiens GN=JUP PE=1 SV=3 - [PLAK_HUMAN] | 18.24 | 16.69 | 5 |
| 21 | P30041 | Peroxiredoxin-6 OS=Homo sapiens GN=PRDX6 PE=1 SV=3 - [PRDX6_HUMAN] | 18.13 | 18.86 | 1 |
| 22 | Q9Y2W1 | Thyroid hormone receptor-associated protein 3 OS=Homo sapiens GN=THRAP3 PE=1 SV=2 - [TR150_HUMAN] | 17.81 | 17.39 | 1 |
| 23 | B4DFG0 | Protein DEK OS=Homo sapiens GN=DEK PE=2 SV=1 - [B4DFG0_HUMAN] | 15.93 | 47.14 | 6 |
| 24 | H7BZJ3 | Thioredoxin (Fragment) OS=Homo sapiens GN=PDIA3 PE=2 SV=1 - [H7BZJ3_HUMAN] | 15.92 | 13.88 | 1 |
| 25 | P67936 | Tropomyosin alpha-4 chain OS=Homo sapiens GN=TPM4 PE=1 SV=3 - [TPM4_HUMAN] | 15.84 | 45.10 | 26 |
| 26 | P52907 | F-actin-capping protein subunit alpha-1 OS=Homo sapiens GN=CAPZA1 PE=1 SV=3 - [CAZA1_HUMAN] | 15.64 | 37.17 | 5 |
| 27 | P30086 | Phosphatidylethanolamine-binding protein 1 OS=Homo sapiens GN=PEBP1 PE=1 SV=3 - [PEBP1_HUMAN] | 15.16 | 8.09 | 1 |
| 28 | P15924 | Desmoplakin OS=Homo sapiens GN=DSP PE=1 SV=3 - [DESP_HUMAN] | 14.97 | 39.39 | 2 |
| 29 | P50395 | Rab GDP dissociation inhibitor beta OS=Homo sapiens GN=GDI2 PE=1 SV=2 - [GDIB_HUMAN] | 14.68 | 30.32 | 6 |
| 30 | P58107 | Epiplakin OS=Homo sapiens GN=EPPK1 PE=1 SV=2 - [EPIPL_HUMAN] | 14.50 | 7.35 | 2 |
| 31 | J3KP15 | Serine/arginine-rich-splicing factor 2 (Fragment) OS=Homo sapiens GN=SRSF2 PE=4 SV=1 - [J3KP15_HUMAN] | 14.29 | 5.83 | 6 |
| 32 | Q96CS3 | FAS-associated factor 2 OS=Homo sapiens GN=FAF2 PE=1 SV=2 - [FAF2_HUMAN] | 13.54 | 11.01 | 1 |
| 33 | E9PAV3 | Nascent polypeptide-associated complex subunit alpha OS=Homo sapiens GN=NACA PE=2 SV=1 - [E9PAV3_HUMAN] | 12.93 | 20.73 | 8 |
| 34 | O14672 | ADA10_HUMAN Disintegrin and metalloproteinase domain-containing protein 10 OS=Homo sapiens OX=9606 GN=ADAM10 PE=1 SV=1 | 12.91 | 25.83 | 2 |
| 35 | P29966 | Myristoylated alanine-rich C-kinase substrate OS=Homo sapiens GN=MARCKS PE=1 SV=4 - [MARCS_HUMAN] | 12.57 | 29.86 | 1 |
| 36 | Q5VU59 | Tropomyosin alpha-3 chain OS=Homo sapiens GN=TPM3 PE=2 SV=1 - [Q5VU59_HUMAN] | 12.21 | 8.43 | 34 |
| 37 | Q9ULV4 | Coronin-1C OS=Homo sapiens GN=CORO1C PE=1 SV=1 - [COR1C_HUMAN] | 11.55 | 16.91 | 11 |
| 38 | Q9HBL7 | Plasminogen receptor (KT) OS=Homo sapiens GN=PLGRKT PE=1 SV=1 - [PLRKT_HUMAN] | 10.92 | 47.24 | 1 |
| 39 | J3KSW8 | Myosin phosphatase Rho-interacting protein (Fragment) OS=Homo sapiens GN=MPRIP PE=4 SV=1 - [J3KSW8_HUMAN] | 9.89 | 46.91 | 6 |
| 40 | P13693 | Translationally-controlled tumor protein OS=Homo sapiens GN=TPT1 PE=1 SV=1 - [TCTP_HUMAN] | 9.28 | 13.54 | 5 |
| 41 | Q02413 | Desmoglein-1 OS=Homo sapiens GN=DSG1 PE=1 SV=2 - [DSG1_HUMAN] | 8.61 | 19.33 | 1 |
| 42 | Q8NBS9 | Thioredoxin domain-containing protein 5 OS=Homo sapiens GN=TXNDC5 PE=1 SV=2 - [TXND5_HUMAN] | 8.01 | 17.95 | 3 |
| 43 | P58546 | Myotrophin OS=Homo sapiens GN=MTPN PE=1 SV=2 - [MTPN_HUMAN] | 7.99 | 6.15 | 2 |
| 44 | H0YL52 | Tropomyosin alpha-1 chain (Fragment) OS=Homo sapiens GN=TPM1 PE=3 SV=1 - [H0YL52_HUMAN] | 7.93 | 16.48 | 31 |
| 45 | O95881 | Thioredoxin domain-containing protein 12 OS=Homo sapiens GN=TXNDC12 PE=1 SV=1 - [TXD12_HUMAN] | 7.02 | 22.05 | 1 |
| 46 | E9PH29 | Thioredoxin-dependent peroxide reductase, mitochondrial OS=Homo sapiens GN=PRDX3 PE=2 SV=1 - [E9PH29_HUMAN] | 7.02 | 10.24 | 2 |
| 47 | B4DT77 | Annexin OS=Homo sapiens GN=ANXA7 PE=2 SV=1 - [B4DT77_HUMAN] | 6.86 | 36.36 | 3 |
| 48 | B7Z1C9 | Chaperonin containing TCP1, subunit 7 (Eta), isoform CRA_a OS=Homo sapiens GN=CCT7 PE=2 SV=1 - [B7Z1C9_HUMAN] | 6.36 | 20.21 | 6 |
| 49 | J3QR44 | Cyclin-dependent kinase 11B OS=Homo sapiens GN=CDK11B PE=4 SV=1 - [J3QR44_HUMAN] | 5.81 | 13.27 | 24 |
| 50 | P09382 | Galectin-1 OS=Homo sapiens GN=LGALS1 PE=1 SV=2 - [LEG1_HUMAN] | 5.75 | 15.71 | 1 |
| 51 | C9J0F2 | Protein-L-isoaspartate(D-aspartate) O-methyltransferase (Fragment) OS=Homo sapiens GN=PCMT1 PE=2 SV=1 - [C9J0F2_HUMAN] | 5.71 | 18.18 | 6 |
| 52 | E9PBS1 | Phosphoribosylaminoimidazole carboxylase (Fragment) OS=Homo sapiens GN=PAICS PE=2 SV=1 - [E9PBS1_HUMAN] | 5.69 | 44.53 | 4 |
| 53 | P55145 | Mesencephalic astrocyte-derived neurotrophic factor OS=Homo sapiens GN=MANF PE=1 SV=3 - [MANF_HUMAN] | 4.84 | 11.83 | 1 |
| 54 | E9PMG4 | Telomerase Cajal body protein 1 OS=Homo sapiens GN=WRAP53 PE=2 SV=1 - [E9PMG4_HUMAN] | 4.79 | 6.19 | 3 |
| 55 | E7EUY0 | DNA-dependent protein kinase catalytic subunit OS=Homo sapiens GN=PRKDC PE=2 SV=1 - [E7EUY0_HUMAN] | 4.79 | 28.43 | 3 |
| 56 | Q15654 | Thyroid receptor-interacting protein 6 OS=Homo sapiens GN=TRIP6 PE=1 SV=3 - [TRIP6_HUMAN] | 4.74 | 16.94 | 2 |
| 57 | Q08554 | Desmocollin-1 OS=Homo sapiens GN=DSC1 PE=1 SV=2 - [DSC1_HUMAN] | 4.55 | 14.94 | 2 |
| 58 | P07996 | Thrombospondin-1 OS=Homo sapiens GN=THBS1 PE=1 SV=2 - [TSP1_HUMAN] | 4.38 | 15.05 | 1 |
| 59 | Q5HYA8 | Meckelin OS=Homo sapiens GN=TMEM67 PE=1 SV=2 - [MKS3_HUMAN] | 4.38 | 4.78 | 2 |
| 60 | Q5TBQ0 | Protein LSM14 homolog B (Fragment) OS=Homo sapiens GN=LSM14B PE=2 SV=1 - [Q5TBQ0_HUMAN] | 4.23 | 23.39 | 4 |
| 61 | J3QRK5 | Protein UBBP4 OS=Homo sapiens GN=UBBP4 PE=4 SV=1 - [J3QRK5_HUMAN] | 4.01 | 5.20 | 22 |
| 62 | K7EKI8 | Periplakin OS=Homo sapiens GN=PPL PE=4 SV=1 - [K7EKI8_HUMAN] | 3.85 | 11.74 | 3 |
| 63 | Q96QK1 | Vacuolar protein sorting-associated protein 35 OS=Homo sapiens GN=VPS35 PE=1 SV=2 - [VPS35_HUMAN] | 3.51 | 19.34 | 1 |
| 64 | Q8TE54 | Anion exchange transporter OS=Homo sapiens GN=SLC26A7 PE=2 SV=2 - [S26A7_HUMAN] | 3.35 | 21.44 | 2 |
| 65 | P02795 | Metallothionein-2 OS=Homo sapiens GN=MT2A PE=1 SV=1 - [MT2_HUMAN] | 3.30 | 23.61 | 6 |
| 66 | B1AKR1 | Calmodulin-like 6, isoform CRA_a OS=Homo sapiens GN=CALML6 PE=2 SV=1 - [B1AKR1_HUMAN] | 3.26 | 6.18 | 2 |
| 67 | P43403 | Tyrosine-protein kinase ZAP-70 OS=Homo sapiens GN=ZAP70 PE=1 SV=1 - [ZAP70_HUMAN] | 3.08 | 52.38 | 3 |
| 68 | Q9H875 | PRKR-interacting protein 1 OS=Homo sapiens GN=PRKRIP1 PE=1 SV=1 - [PKRI1_HUMAN] | 3.04 | 14.47 | 1 |
| 69 | Q15019 | Septin-2 OS=Homo sapiens GN=SEPT2 PE=1 SV=1 - [SEPT2_HUMAN] | 3.04 | 34.48 | 11 |
| 70 | Q96IZ0 | PRKC apoptosis WT1 regulator protein OS=Homo sapiens GN=PAWR PE=1 SV=1 - [PAWR_HUMAN] | 3.00 | 8.52 | 1 |
| 71 | Q9Y2T2 | AP-3 complex subunit mu-1 OS=Homo sapiens GN=AP3M1 PE=1 SV=1 - [AP3M1_HUMAN] | 2.90 | 29.66 | 1 |
| 72 | D6RBE9 | Annexin OS=Homo sapiens GN=ANXA5 PE=2 SV=1 - [D6RBE9_HUMAN] | 2.86 | 24.03 | 4 |
| 73 | J3KRE2 | Rho GDP-dissociation inhibitor 1 OS=Homo sapiens GN=ARHGDIA PE=4 SV=1 - [J3KRE2_HUMAN] | 2.83 | 19.62 | 4 |
| 74 | B4DDF4 | Calponin-2 OS=Homo sapiens GN=CNN2 PE=2 SV=1 - [B4DDF4_HUMAN] | 2.61 | 19.65 | 5 |
| 75 | J3KSC4 | Ras-related C3 botulinum toxin substrate 3 (Fragment) OS=Homo sapiens GN=RAC3 PE=3 SV=1 - [J3KSC4_HUMAN] | 2.54 | 6.64 | 9 |
| 76 | F8WA86 | Calponin-3 OS=Homo sapiens GN=CNN3 PE=2 SV=1 - [F8WA86_HUMAN] | 2.45 | 5.70 | 4 |
| 77 | B4DH42 | E3 ubiquitin-protein ligase HECW1 OS=Homo sapiens GN=HECW1 PE=2 SV=1 - [B4DH42_HUMAN] | 2.33 | 2.81 | 2 |
| 78 | F8WF16 | Periphilin-1 OS=Homo sapiens GN=PPHLN1 PE=2 SV=1 - [F8WF16_HUMAN] | 2.28 | 12.54 | 10 |

Note: Red: Kir2.1; Blue: Adam10; Yellow: proteins associated with invasion and metastasis based on the literature mining.
